# Rethinking the AI Paradigm for Solubility Prediction of Drug‑Like Compounds with Dual‐Perspective Modeling and Experimental Validation

**DOI:** 10.1002/advs.202511667

**Published:** 2025-09-25

**Authors:** Qilin Zhu, Yuxin Qiu, Guzhong Chen, Wenyao Chen, Xiang Zhang, Zhiwen Qi, Xuezhi Duan, De Chen, Zhen Song

**Affiliations:** ^1^ State Key Laboratory of Chemical Engineering School of Chemical Engineering East China University of Science and Technology 130 Meilong Road Shanghai 200237 China; ^2^ Department of Chemical Engineering Columbia University New York NY 10027 USA; ^3^ Department of Chemical Engineering Norwegian University of Science and Technology Trondheim 7034 Norway

**Keywords:** applicability domain, artificial intelligence, drug discovery, dual‐perspective modeling

## Abstract

Aqueous solubility is a crucial property for drug development, as it not only influences the drug delivery process but also determines the bioavailability of drugs. However, solubility prediction remains a formidable challenge, even after decades of research. Most previously‐reported machine learning (ML) models generalize poorly on external sets due to the vast chemical space of drug compounds. In this report, the largest aqueous solubility dataset of drug and drug‐like molecules so far is compiled, based on which reliable models for drug solubility prediction are developed by comparative modelling with assorted regression and classification algorithms. Under current circumstances, even advanced deep learning models are found less accurate than the stacking of multiple statistical ML algorithms due to data limitation. Analysis of applicability domain further verifies the generalization capability of the models for the drug domain, based on which the entries without experimental solubility in the DrugBank database are populated and categorized. Finally, the solubility of ten potential drug molecules is experimentally determined for the first time, again revealing the high reliability of our models. Hence, this work is believed to provides a comprehensive benchmark for future solubility prediction models and a powerful tool to guide new drug discovery.

## Graphical abstract

Aqueous solubility governs drug delivery and bioavailability, yet its prediction remains challenging. Utilizing the largest curated dataset of drug‐like molecules, prediction models are constructed through dual‐perspective benchmarking. Stacking methods outperform advanced deep learning due to data constraints. These validated models enabled systematic annotation of DrugBank solubility profiles; experimental determination for ten novel compounds further demonstrated their reliability.

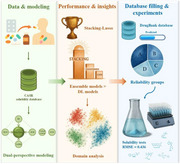

## 1 Introduction

In novel drug discovery, screening of molecular properties helps quickly eliminate candidate drug compounds that do not meet clinical requirements, dramatically reducing the time and cost associated with drug development. As a vital physiochemical property, aqueous solubility determines the in vivo dissolution ability of drugs and governs their bioavailability.^[^ ^1^, ^2^ ^]^ Moreover, sufficient solubility is essential to facilitate in vitro clinical trials.^[^ ^3^, ^4^, ^5^ ^]^ Unfortunately, solubility is not a well‐documented property, with only a limited number of chemicals having recorded solubility values in public databases (e.g., PubChem). Consequently, solubility values for novel compounds must be obtained either by experiments or through computational methods. While experimental approaches are reliable and accurate, their high costs, due to expensive potential drug compounds and lengthy experimental procedures, hinder high‐throughput screening. Thus, reliable computational methods are invaluable for estimating solubility and prioritizing candidate drugs.

One of the classic computational approaches to calculate aqueous solubility is the general solubility equation (GSE),^[^ ^6^ ^]^ which is a simple QSPR model based on thermodynamics using only two descriptors: melting point and the octanol–water partition coefficient (Log P). However, this method is typically employed to predict the aqueous solubility of solid non‐electrolytes and is not applicable to compounds with unknown experimental melting points and Log P.^[^ ^7^, ^8^ ^]^ Alternative approaches, such as Hansen^[^ ^9^ ^]^ and Hildebrand^[^ ^10^ ^]^ solubility parameters, avoid the use of empirical parameters but are limited to providing relative solubility.^[^ ^11^ ^]^ Other first‐principles methods including the Flory‐Huggins paramete,r^[^ ^12^ ^]^ molecular dynamics^[^ ^13^ ^]^ and Monte Carlo simulations^[^ ^14^ ^]^ require long computational times, often ranging from 6 to 24 h per molecule.^[^ ^15^ ^]^ The conductor‐like screening model for realistic solvation (COSMO‐RS),^[^ ^16^, ^17^, ^18^ ^]^ as a hybrid quantum chemistry‐statistical thermodynamic method, necessitates *σ*‐profile inputs from time‐consuming density functional theory (DFT) calculations and has a notably varied performance from case to case owing to its fully predictive character.

Recent advances in solubility prediction largely rely on quantitative structure property relationship (QSPR) models driven by statistical machine learning (ML). Ghanavati et al. developed a ML framework employing XGBoost with 2D and 3D Mordred descriptors, achieving performance with R^2^ = 0.918 and RMSE = 0.613 across four datasets including ESOL, AQUA, PHYS, and OCHEM.^[^ ^19^ ^]^ Tayyebi et al. demonstrated the effectiveness of Random Forest models using Morgan fingerprints, reporting R^2^ values of 0.880 and RMSE values of 0.640 on 8,438 compounds from three literature‐based datasets (Vermeire, Boobier, and Delaney datasets).^[^ ^20^ ^]^ As a prevailing trend, more and more researchers started to employ deep learning (DL) for aqueous solubility prediction. Panapitiya et al. implemented different DL modeling approaches (fully connected neural networks, recurrent neural networks, graph neural networks (GNNs), and SchNet) to predict solubility from several molecular representations, finally achieving R^2^ = 0.920 and RMSE = 0.600 on the Delaney dataset.^[^ ^21^ ^]^.Cui et al. explored deep ResNets with 14, 20, and 26 layers, achieving R^2^ values of 0.720–0.790 and RMSE values of 0.988–1.151 for 10‐fold cross‐validation on the ChemIDplus database.^[^ ^22^ ^]^ Francoeur and Koes developed SolTranNet, a molecule attention transformer, achieving RMSE of 1.459 on AqSolDB and 1.711 on a withheld test set.^[^ ^23^ ^]^ With more relevant works summarized in Table S1 (Supporting Information), it can be found that current ML and DL approaches predominantly rely on limited training sets, which may compromise model generalizability. Moreover, the predominant trend favors the adoption of complex DL models, often without rigorous comparative evaluation against conventional ML baselines. While some models have demonstrated excellent accuracy (e.g., RMSE < 0.7) on internal training datasets, they often perform poorly on external datasets due to limited generalizability and ill‐defined applicability domains (AD), particularly for drug‐like molecules that are frequently underrepresented during training. Additionally, most existing studies lack thorough AD analysis regarding their alignment with drug‐like chemical space and rarely include experimental validation of predictions, raising concerns about the reliability of these models in guiding drug design.

This study compiles an up‐to‐date most comprehensive aqueous solubility dataset, enabling the rigorous development of ML models for both regression and classification tasks. Importantly, organic salts, inorganic molecules, and metal‐containing compounds are considered in training and external tests. A wide range of methods are explored, including statistical ML algorithms, stacking models, and advanced DL architectures such as Transformers and GNNs. Surprisingly, the results show that a stacking model using Lasso regression outperforms both its base learners and DL models, surpassing all reference models for drug solubility prediction. For classification tasks, LightGBM emerges as the best‐performing algorithm. Moreover, the findings reveal that regression and classification models complement one another rather than serving as substitutes.

To further enhance the utility of these models, the AD of the training set is analyzed and compared to the chemical space of potential drug molecules in the DrugBank database (version 5.1.12),^[^ ^24^ ^]^ which lack recorded solubility values. These empty entries are populated with predicted solubility values and classes, and categorized into different reliability groups based on their AD status and consistency across models. Finally, the predictive capability of the models is experimentally validated on a list of these latent drug candidates, demonstrating their high reliability and practical applicability.

## 2 Results and Discussion

### 2.1 Data Curation

To construct a comprehensive dataset on aqueous solubility, extensive data were compiled from various literature and databases,^[^ ^25^, ^26^, ^27^, ^28^, ^29^, ^30^, ^31^, ^32^, ^33^, ^34^, ^35^, ^36^, ^37^, ^38^, ^39^ ^]^ including the recently accessible Wiki‐ps0 database. The resulting dataset, referred to as Comprehensive Aqueous Solubility Repository (CASR‐1), retains only experimental aqueous solubility data recorded at room temperature (25 ± 2 °C). All solubility values were converted to the base‐10 logarithm of moles per liter (Log S) and solubility class (see methods section) for classification tasks. Unlike previous studies, CASR‐1 includes salts, inorganic compounds, and molecules with metal elements to ensure a broader applicability. Entries of single atom SMILES strings and ions (charge not equal to 0) were excluded. For compounds lacking SMILES strings, other identifiers such as CAS numbers were utilized to acquire SMILES strings from PubChem. Entries without sufficient information were excluded.

**Figure 1:**
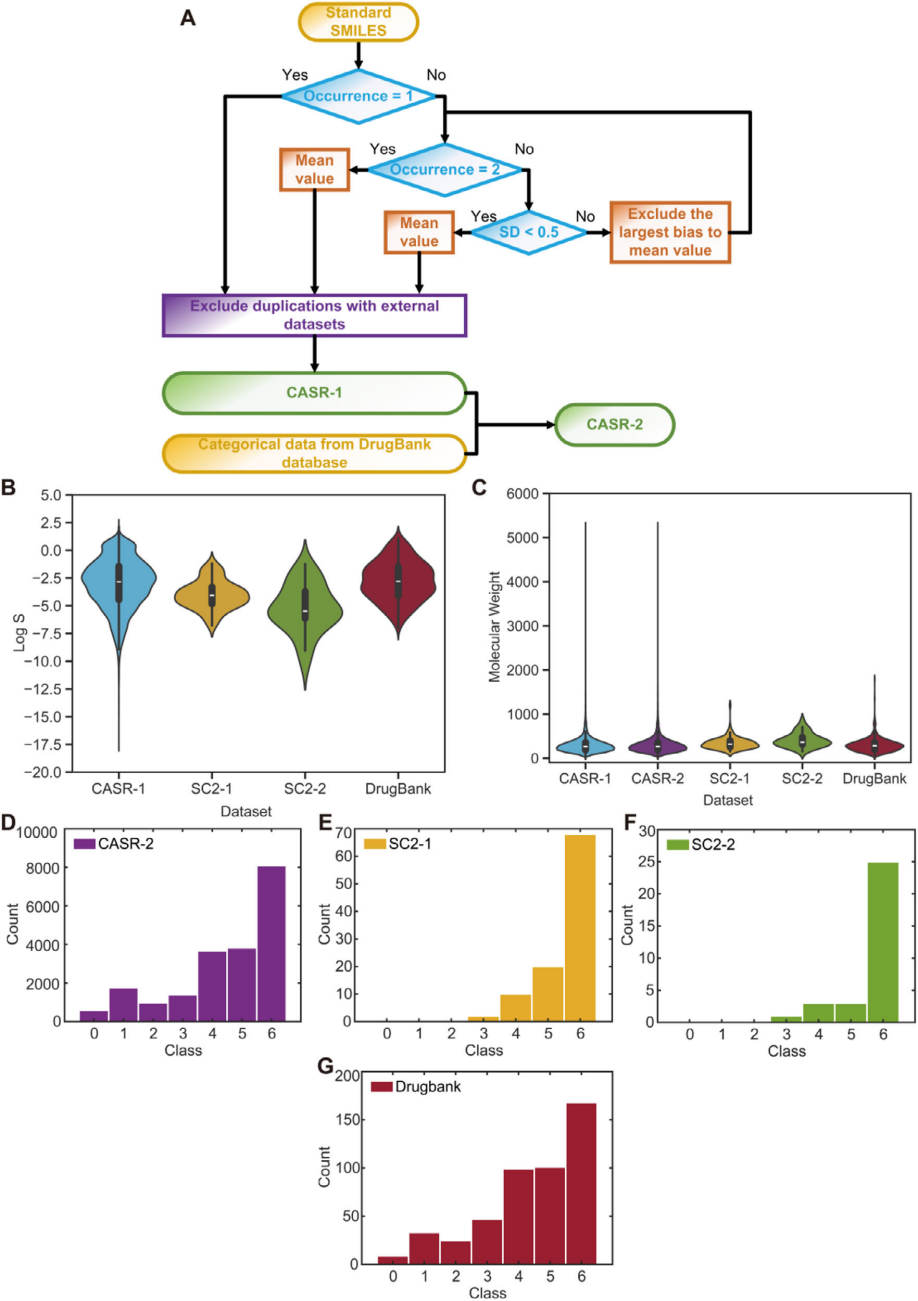
Datasets in this work and their distributions. A) Workflow of data curation for CASR. B–(C) Logarithm solubility distribution and molecular weight distribution of CASR and external datasets. D–G) Solubility class distribution of CASR‐2, SC2‐1, SC2‐2, and DrugBank (‘0’–‘6’ represent ‘very soluble’, ‘freely soluble’, ‘soluble’, ‘sparingly soluble’, ‘slightly soluble’, ‘very slightly soluble’, ‘practically insoluble or insoluble’, respectively, according to European Pharmacopoeia).

To validate SMILES strings, Python RDKit package was used to convert SMILES to molecular formats. Invalid SMILES strings, flagged during this process, were either corrected using PubChem or removed. Duplicate molecules were merged using a protocol similar to that of Sorkun et al.^[^ ^25^ ^]^ (**Figure** **1**A), where SMILES strings were standardized with RDKit to ensure consistent representation. If a molecule is only recorded once or twice, the solubility value or the average was simply accepted by CASR‐1. For molecules with multiple records, average values were used unless the standard deviation exceeds 0.5 Log unit (usually regarded as the average experimental errors in solubility determination^[^ ^40^ ^]^), in which case outliers would be removed iteratively. After applying this criterion, 390 entries with data from three or more sources resulted in an average SD of 0.31 Log unit, with detailed information summarized in Supplementary data.xlsx file (worksheet ‘SD’). Molecules with Log S differences smaller than 0.01 were treated as single occurrences. It is noteworthy that ≈95% of the molecules in the training set comply with Lipinski's ‘Rule of Five’ (molecular weight < 500, calculated Log *p* < 5, number of H‐bond donors < 5, and number of H‐bond acceptors < 10),^[^ ^41^, ^42^ ^]^ a widely recognized criterion for drug‐likeness, and can thus be considered drug‐like. While many molecules in our datasets are not themselves pharmaceuticals in current use, they exhibit drug‐like characteristics and occupy a chemical space similar to that of approved drugs as well as prospective drug candidates.

External test sets, including the DrugBank database (version 5.1.12) and the *Second Solubility Challenge* dataset^[^ ^43^ ^]^ (SC2), were utilized to evaluate the models. The DrugBank dataset contains Food and Drug Administration (FDA)‐approved drugs and experimental candidates, categorized into subsets based on the availability of solubility values (DrugBank set) or mere solubility class (DrugBank‐class). The remaining entries without experimental solubility values were termed DrugBank‐vacant. The SC2 dataset, organized by the American Chemical Society in 2019, includes two subsets: SC2‐1 (tight solubility measurements; SD = 0.17) and SC2‐2 (loose measurements; SD = 0.62).^[^ ^44^ ^]^ Further, to make full use of all accessible data, DrugBank‐class were merged with CASR‐1 to construct the training set for classification, namely CASR‐2.

To prevent data leakage, overlapping compounds between CASR and external test sets were removed. Molecular weight and Log S distributions for CASR shows near‐normal trends, with most compounds falling in the molecular weight range of 200–300 and Log S range between −5 and −2 (Figure 1B,C; Figures S1–S3, Supporting Information). However, solubility classes are imbalanced, with a predominance of poorly soluble compounds (classes ‘5’ and ‘6’; Figure 1D–G). **Table** **1** summarizes the datasets used in this study.

**Table 1:** Datasets involved in this work, compared with those in reference works.

Datasets^a)^	Number of Molecules	Log S
**CASR‐1**	19 942	−17.5 to 2.1
**CASR‐2**	20 266	/
**SC2‐1**	100	−6.8 to −1.2
**SC2‐2**	32	−10.4 to −1.2
**DrugBank**	482	−7.0 to 1.1
Panapitiya et al.^[^ ^21^ ^]^	17 149	−17.5 to 1.7
Tang et al.^[^ ^58^ ^]^	1310	−11.6 to 1.6
Wang et al.^[^ ^30^ ^]^	1640	−11.6 to 1.6
Delaney et al.^[^ ^31^ ^]^	1100	−11.6 to 1.6
Huuskonen et al.^[^ ^59^ ^]^	1011	−11.6 to 1.6
Cui et al.^[^ ^22^ ^]^	9979	−18.2 to 1.7

^a)^ Datasets used in this work are in **bold**.

### 2.2 Machine Learning Methods and Evaluation Metrics

Four statistical ML algorithms were employed as base learners: Random Forest (RF), Extreme Gradient Boosting (XGB), Support Vector Machine (SVM), and LightGBM, which have been widely used for drug toxicity prediction,^[^ ^45^ ^]^ environmental science,^[^ ^46^ ^]^ polymer science,^[^ ^47^ ^]^ and material informatics.^[^ ^48^ ^]^ These models were combined using stacking ensembles^[^ ^49^ ^]^ with meta‐learners including Multiple Linear Regression (MLR), Lasso, and Ridge regression (for classification tasks, Logistic Regression (LR) was deployed as meta‐learner). Additionally, an automatic machine learning (AutoML) framework in our previous research^[^ ^50^ ^]^ was also applied. In this framework, the features are input to several base models for multi‐layer stackings with default hyperparameters. The framework automatically searches for the base‐learners, meta‐learners and number of layers of stacking that output the best prediction accuracy.

For comparison, two advanced DL models, namely Transformer‐convolutional neural networks (Transformer‐CNN)^[^ ^51^ ^]^ and GNN,^[^ ^52^ ^]^ were implemented to generate learned molecular representations directly from raw inputs, such as SMILES strings and molecular graphs, without relying on predefined descriptors. These DL methods autonomously learn structural and physicochemical features from data, offering an alternative to traditional descriptor‐based approaches. Moreover, unlabeled pre‐training approaches, such as the BERT Masked Language Model (MLM), are applicable to Transformer‐based models.^[^ ^53^ ^]^ This enables them to learn molecular structural information more comprehensively, thereby achieving higher prediction accuracy on downstream fine‐tuning tasks. Numerous studies have demonstrated that Transformer‐based models achieve significantly superior accuracy in molecular property prediction compared to traditional machine learning methods.^[^ ^54^, ^55^, ^56^, ^57^ ^]^

To evaluate the performance of all models, a comprehensive set of metrics was employed. For regression tasks, the evaluation includes R^2^ (coefficient of determination), root mean square error (RMSE), and mean absolute percentage deviation (MAPD), along with two additional metrics^[^ ^32^ ^]^ namely %Log S ± 0.7 and %Log S ± 1.0, to account for model accuracy and applicability across different solubility ranges. These additional metrics better reflect performance extremes and practical model utility, respectively. For classification tasks, performance was assessed using accuracy, precision, recall, F1 score, the area under the Receiver Operating Characteristic (ROC) curve (AUC), and average precision (AP). To provide a comprehensive assessment, the best‐performing model for each metric received one point, contributing to a cumulative ‘regression/classification score’ for ranking purposes. The model with the highest overall score was selected as the optimal model for each task, ensuring robust and reliable predictions across both internal and external datasets.^[^ ^53^ ^]^

### 2.3 Feature Engineering

**Figure 2:**
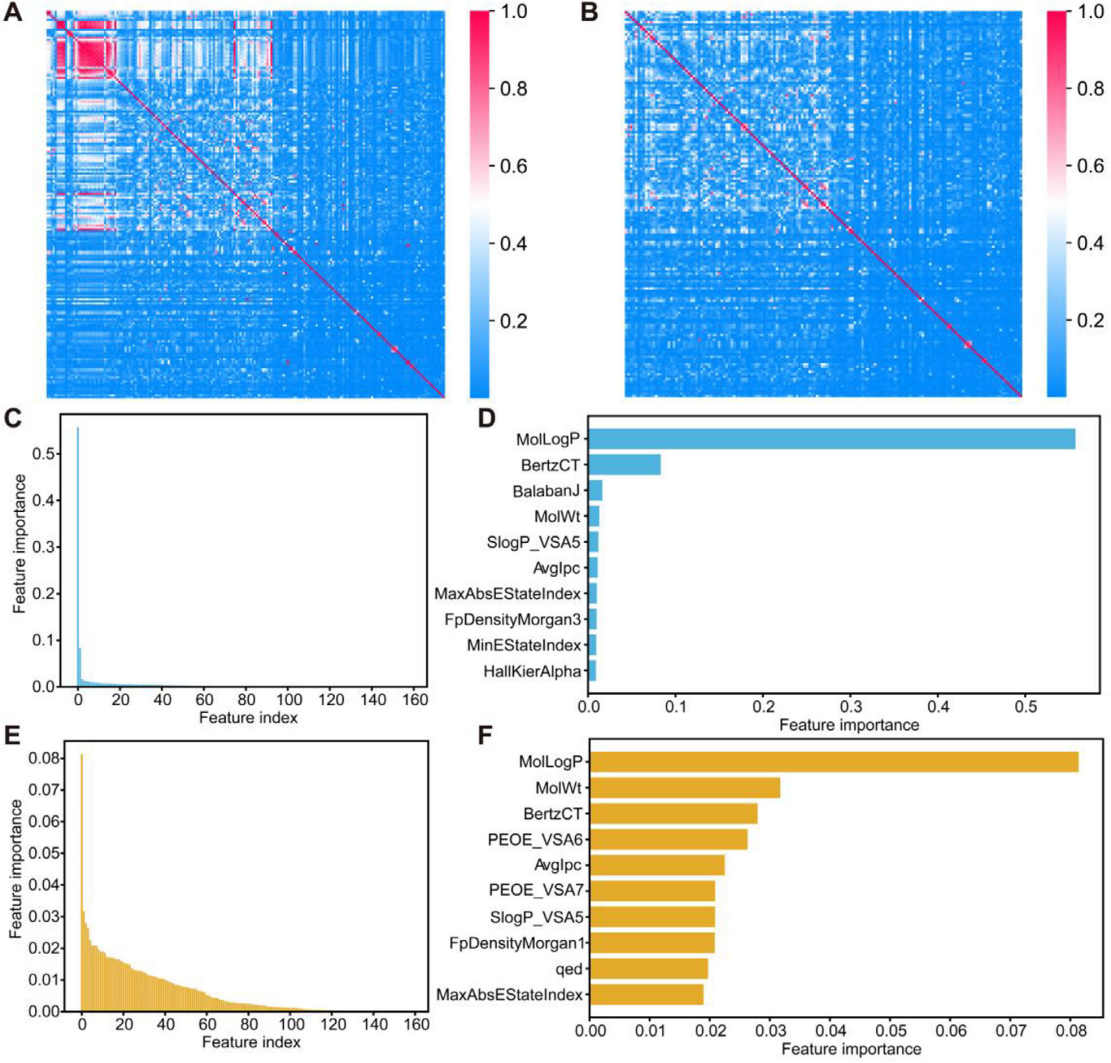
Feature selection for regression and classification modelling. A, B) Correlation between features before and after removal of highly correlated features. C, D) Importance of all features and top 10 most important features in regression task calculated by Random Forest algorithm. E, F) Importance of all features and top 10 most important features in classification task calculated by Random Forest algorithm.

Feature representations or descriptors play a critical role in the predictive performance of ML models. In this work, RDKit descriptors^[^ ^60^ ^]^ were used to represent the structure and physicochemical properties of compounds for building base models and stacking models. The RDKit package provides 209 descriptors, capturing a broad range of aspects including molecular properties, topological information, connectivity, and constitution.^[^ ^61^ ^]^ For compounds containing metal elements, 12 descriptors could not be generated and were excluded from analysis (Table S2, Supporting Information). To mitigate overfitting and redundancy, descriptors with a correlation coefficient |R| > 0.9 were removed (**Figure** **2**A–B), resulting in a refined set of 159 descriptors, which were subsequently standardized. Feature importance was then assessed using the Random Forest algorithm for both regression and classification tasks (Figure 2C–F). For regression tasks, all 159 features were kept because there is a clear decreasing tendency of predictive accuracy as declining the number of features (Table S3, Supporting Information); for classification, features with importance scores below 0.01were excluded (Table S4, Supporting Information). Notably, MolLogP emerges as the most significant feature in both tasks. In regression, MolLogP accounts for nearly half of the predictions, whereas in classification, its contribution is ≈8%, indicating a more balanced importance distribution.

Interestingly, six descriptors, including MolLogP, appear in the top 10 for both tasks. This overlap highlights their consistent relevance to solubility prediction. However, differences in feature importance between regression and classification tasks suggest that while regression relies on a combination of dominant and supporting features, classification performance is driven by a smaller set of equally significant descriptors. This distinction underscores the complementary nature of regression and classification models, as further discussed in subsequent sections.

### 2.4 Regression Tasks

**Figure 3:**
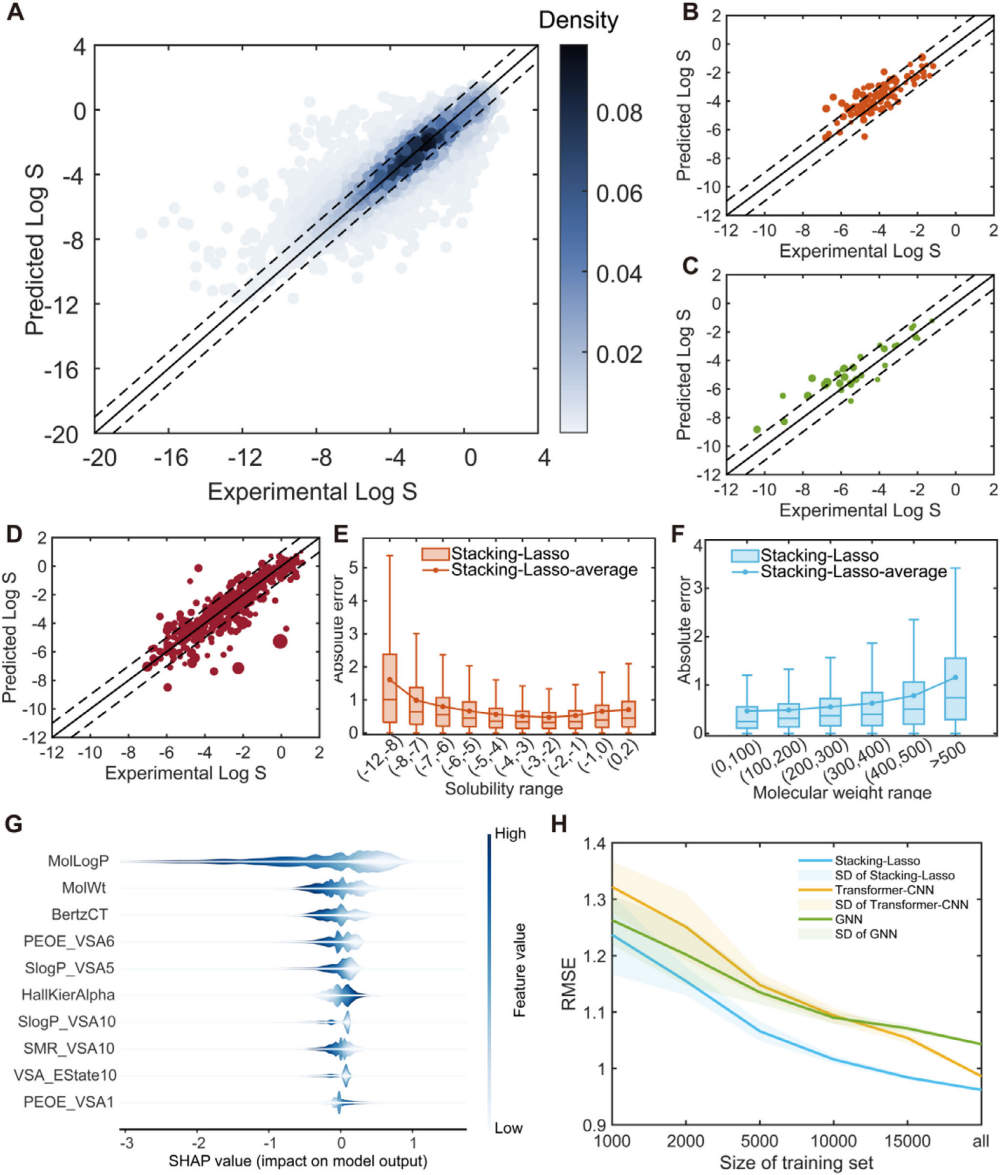
Results of regression task from Stacking‐Lasso model. A–D) Experimental Log S vs predicted Log S for 5‐fold internal cross‐validation and external tests (SC2‐1, SC2‐2, and DrugBank, respectively). The results of external tests are averages of five repetitions, and the corresponding SD is expressed by the sizes of data points. Two dashed lines represent range of Log S ± 1.0. E, F) Influence of solubility value and molecular weight on regression performance for 5‐fold cross‐validation (outliers are hidden so as to ensure the clarity of the figures). G) SHAP analysis for Stacking‐Lasso regression model. H) Effect of changes on training data size to models’ performance, evaluated through internal cross‐validation.

The 5‐fold cross‐validations are performed to identify optimal hyperparameters (Table S5, Supporting Information), after which models are trained and tested. The results are summarized in Tables S6 and S7, and Figures S4–S12 (Supporting Information). The performance among the four base models—RF, XGB, LightGBM, and SVR—remains almost consistent across both internal cross validation and external tests. All stacking models (Stacking‐MLR, Stacking‐Ridge, Stacking‐Lasso, and AutoML) demonstrate superior predictive accuracy compared to the base models and DL models (GNN and Transformer‐CNN). Notably, the DL models demonstrate relatively less competitive performance on the test sets when compared to all the statistical ML models. The high correlations between predictions of the three stacking models suggest limited room for further improvement through additional stacking (Figures S13 and S14, Supporting Information). Overall, the stacking model with Lasso as meta‐learner exhibits the best performance on all the five metrics of RMSE, R^2^, %Log S ± 0.7, %Log S ± 1.0, and MAPD. Experimental versus predicted Log S plots (**Figure** **3**A–D) reveal that most points fall within the Log S ± 1.0 range, demonstrating the model's reliability and potential for practical drug development applications.

To gain deeper insights into the contributions of individual features, SHapley Additive exPlanations (SHAP) analysis was performed for the Stacking‐Lasso model (Figure 3G; Figure S15, Supporting Information). The results indicate that five features (‘MolLogP’, ‘MolWt’, ‘BertzCT’, ‘SlogP_VSA5’, and ‘HallKierAlpha’) consistently rank among the top 10 most influential descriptors. MolLogP, representing the base‐10 logarithm of the octanol/water partition coefficient, emerges as the most impactful feature, exhibiting a strong negative correlation with predicted solubility. This trend aligns with our chemistry domain knowledge, as higher hydrophobicity typically reduces solubility in aqueous environments. Other notable features include molecular weight (MolWt), which shows a negative correlation with solubility, likely due to the reduced proportion of hydrophilic groups in larger molecules. BertzCT and SlogP_VSA5, representing molecular complexity and polar surface area, respectively, are also negatively correlated with solubility. In contrast, HallKierAlpha, a measure of molecular polarity, demonstrates a positive correlation, consistent with the principle that solutes dissolve more readily in solvents with similar polarity—water being a highly polar solvent.

The trends of variation between prediction errors and solubility or molecular weight are consistent with previous work.^[^ ^21^ ^]^ Relatively larger errors are observed for molecules with extremely high or low Log S values (Figure 3E). This can be attributed to data scarcity in these regions (Figure 1C) and greater experimental uncertainty in measuring low concentrations. Accurately measuring extremely low concentrations is challenging,^[^ ^62^ ^]^ resulting in greater experimental errors in solubility measurements for molecules with low solubility compared to those with higher solubility. Moreover, calculated Log P with extreme magnitudes are often inaccurate and show weak correlation with Log S at these extreme values.^[^ ^63^ ^]^ As illustrated in Figure S16 (Supporting Information), changes in Log P beyond these thresholds do not significantly influence predicted Log S. These factors contribute to the disproportionately large prediction errors observed for low‐solubility molecules. On the other hand, despite significant Log S errors for low‐solubility molecules, the actual solubility errors (mol/L or g/L, without logarithm) remain minimal, as evidenced by high classification scores for class ‘6’ in the classification task (see next section). For compounds with molecular weight below 300, prediction errors consistently stabilize ≈0.5 (Figure 3F). However, for larger molecules, prediction errors escalate significantly, possibly due to increased structural complexity and data scarcity for molecules with high molecular weights.

Interestingly, the stacking models using traditional ML algorithms outperform DL models. This result is unexpected, as DL approaches are designed to autonomously extract features from raw inputs. The likely explanation is that the training set size remains insufficient for DL methods to generalize effectively for drug solubility prediction. Overfitting may occur when structural information for complex, drug‐like molecules is incomplete. In contrast, thermodynamic descriptors like MolLogP, which are highly correlated with solubility, allow traditional QSPR approaches to more effectively capture solubility relationships. Nevertheless, the underperformance of the DL models as opposed to the stacking models is likely to be overturned by adding more data in the training set. As evidenced in Figure 3H, the predictive accuracy increases for all three models (Stacking Lasso, Transformer‐CNN, and GNN) as the size of training set grows, but the Transformer‐CNN presents a tendency to outperform the Stacking‐Lasso if more data are provided. As for GNN, the trend of accuracy change is nearly the same as the Stacking‐Lasso, suggesting their possibly similar predictive capacity in a larger dataset.

Finally, the performance of the Stacking‐Lasso model is compared to other reference prediction models (**Table** **2**, Table S8, Supporting Information). The results clearly demonstrate that the Stacking‐Lasso model outperforms benchmark ML methods and some classic computational approaches (GSE, Abraham solubility equation (ASE), and COSMO‐RS) across all the external test sets, highlighting its superior generalization capability for drug solubility prediction. Best performance on SC2‐1 is from the work by Avdeef,^[^ ^39^ ^]^ trained on the Wiki‐ps0 database, which consists of high quality intrinsic solubility values. Even trained just with the fundamental RF algorithm, optimal results were obtained. Hence, data quality is another crucial factor affecting model accuracy. However, the SC2 set is from the Wiki‐ps0 database and naturally comes from the so called ‘same distributions’. Moreover, trained on a small set of ≈3000 molecules, the AD is not enough to cover the chemical space of potential drugs. As for COSMO‐RS, the default QSPR parameters for its iterative method in solubility calculation were fitted by 150 drug‐like molecules.^[^ ^16^ ^]^ These parameters need to be refitted for more accurate drug solubility prediction across a broader space.

**Table 2:** Performance comparison between other reference methods with Stacking‐Lasso model developed in this work by RMSE.^a)^

Method		SC2‐1	SC2‐2	DrugBank
Reference machine learning methods	Ours (Stacking‐Lasso)	0.801	1.029	0.909
	Tosca et al.^[^ ^64^ ^]^ (ANN)	0.970	1.180	/
	Panapitiya et al.^[^ ^21^ ^]^ (MDM)	0.910	1.170	/
	Ramos et al.^[^ ^65^ ^]^ (Deep ensemble LSTM)	0.983	1.205	/
	Francoeur et al.^[^ ^23^ ^]^ (Molecule attention transformer)	0.952	1.243	1.067
	Avdeef^[^ ^39^ ^]^ (RF)	0.750	1.050	/
	Tetko et al.^[^ ^66^ ^]^ (ANN)	1.042	1.706	1.029
Other approaches	GSE^[^ ^64^ ^],^ ^b)^	1.121	1.199	1.579
	ASE	4.065	3.118	5.936
	Modified ASE^c)^	1.251	1.245	1.473
	COSMO‐RS^d)^	/	/	1.341

^a)^ For machine learning methods, if metrics for external tests are not presented in source literature, we ran the provided models to obtain the RMSE values. When there is no way to acquire the RMSE value, the item is filled with ‘/’;

^b)^ GSE results for SC2‐1 and SC2‐2 is from literature;

^c)^ Coefficients are refitted using CASR‐1;

^d)^ The structure optimization for 31 out of 482 molecules in DrugBank set failed, so this is the result from calculations for 451 molecules (see methods and Supporting Information). Considering that most molecules with failed optimization are complex molecules or ones with rare elements, which are hard to predict, this RMSE of 1.341 is even an overestimation.

### 2.5 Classification Tasks

**Figure 4:**
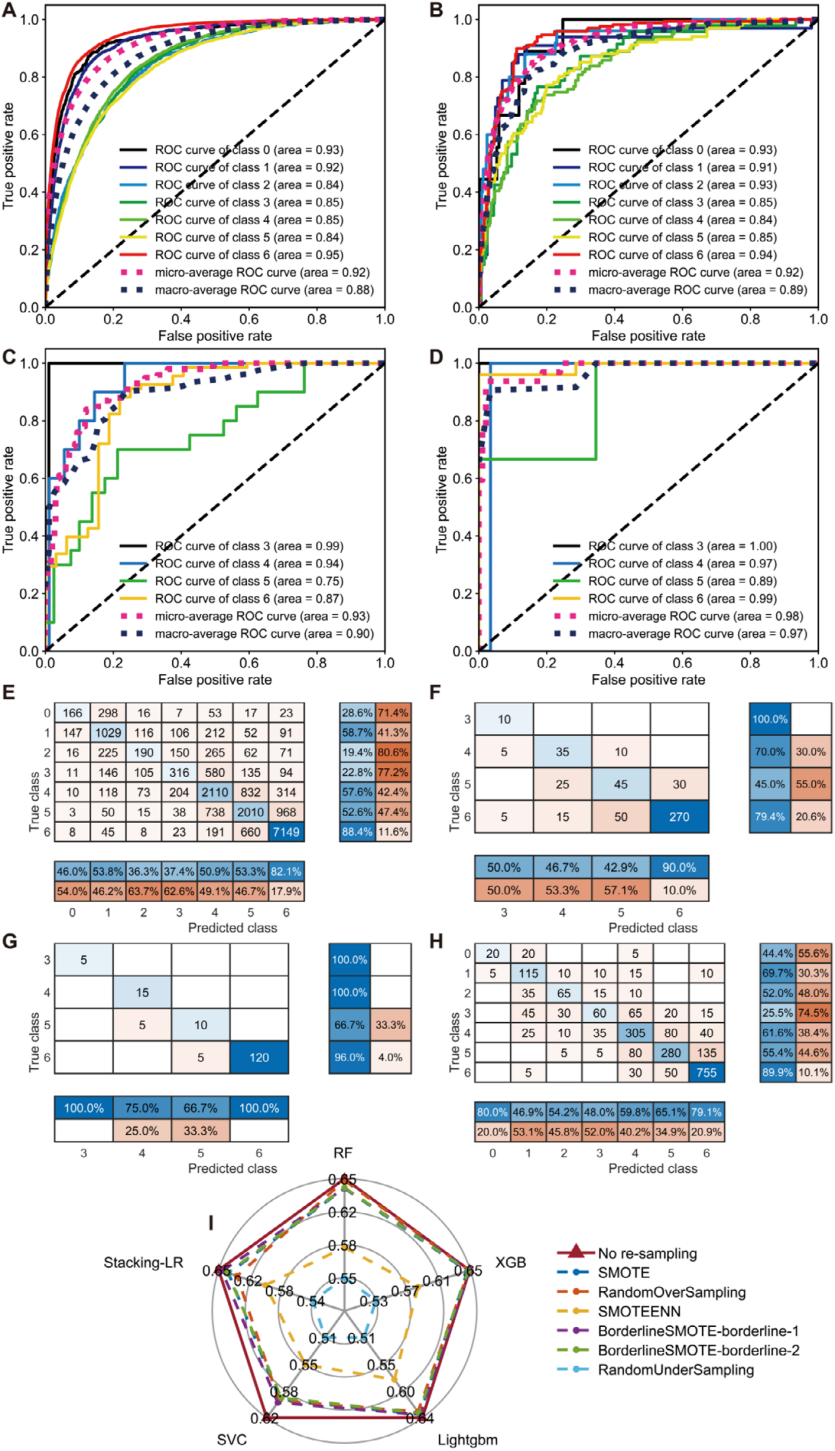
Results of classification task from LightGBM model. (A–D) ROC curves for 5‐fold internal cross‐validation and external tests (SC2‐1, SC2‐2, and DrugBank, respectively). The results of external tests are totals of five repetitions. E–H) Confusion matrices for 5‐fold internal cross‐validation and external tests (SC2‐1, SC2‐2, and DrugBank, respectively). The results of external tests are totals of five repetitions. I) Effect of data re‐sampling on model performance, denoted by total results of 5‐fold internal cross‐validation with the metric ‘accuracy’.

Similar to regression tasks, the 5‐fold cross‐validations were employed for hyperparameter optimization in classification modelling (Table S9, Supporting Information) and models were trained on the optimized parameters. While the stacking model with Logistic Regression as the meta‐learner performs best during internal cross‐validation, LightGBM achieves the highest accuracy on external datasets, including SC2‐1, SC2‐2, and DrugBank (Tables S10 and S11, Figure S17–21, Supporting Information). Consequently, LightGBM was selected as the final model for populating the DrugBank database. Classification accuracy is particularly high for compounds with low solubility (class ‘6’) across both internal and external sets. However, highly soluble compounds are more challenging to classify accurately, as reflected by relatively low AUC values in the ROC curves (**Figure** **4**A–D) as well as low precision and recall in confusion matrices for classes ‘0’ through ‘5’ (Figure 4E–H). To investigate whether data imbalance in the training set contributed to these errors, six data re‐sampling techniques^[^ ^67^, ^68^, ^69^, ^70^, ^71^, ^72^ ^]^ were applied to balance the training set (the test set of each fold is free from data re‐sampling). However, no significant improvements are observed, and in some cases, model performance even deteriorates (Figure 4I; Table S12, Supporting Information). These results suggest that errors stem primarily from the insufficient data on high‐solubility molecules (Figure 1D) rather than imbalances in the training set.

When converting predicted solubility values from Stacking‐Lasso regression model into solubility class, classification accuracy was found to be inferior to that of the LightGBM classification model (**Table** **3**). Additionally, classification models occasionally produce large biases, such as predicting class ‘6’ for true class ‘1’, a scenario less likely with regression models. These findings highlight the complementary nature of regression and classification models. While regression models excel in fine‐grained predictions, classification models provide broader categorical insights, making them effective supplements and validations for one another.

**Table 3:** Classification results of converting the predicted values from Stacking‐Lasso regression model to solubility class.

Database	Accuracy	Precision	Recall	F1
**SC2‐1**	0.700	0.486	0.435	0.453
**SC2‐2**	0.875	0.813	0.767	0.781
**DrugBank**	0.602	0.431	0.418	0.422
**All**	0.632	0.431	0.417	0.422

### 2.6 Applicability Domain Analysis and Database Population

**Figure 5:**
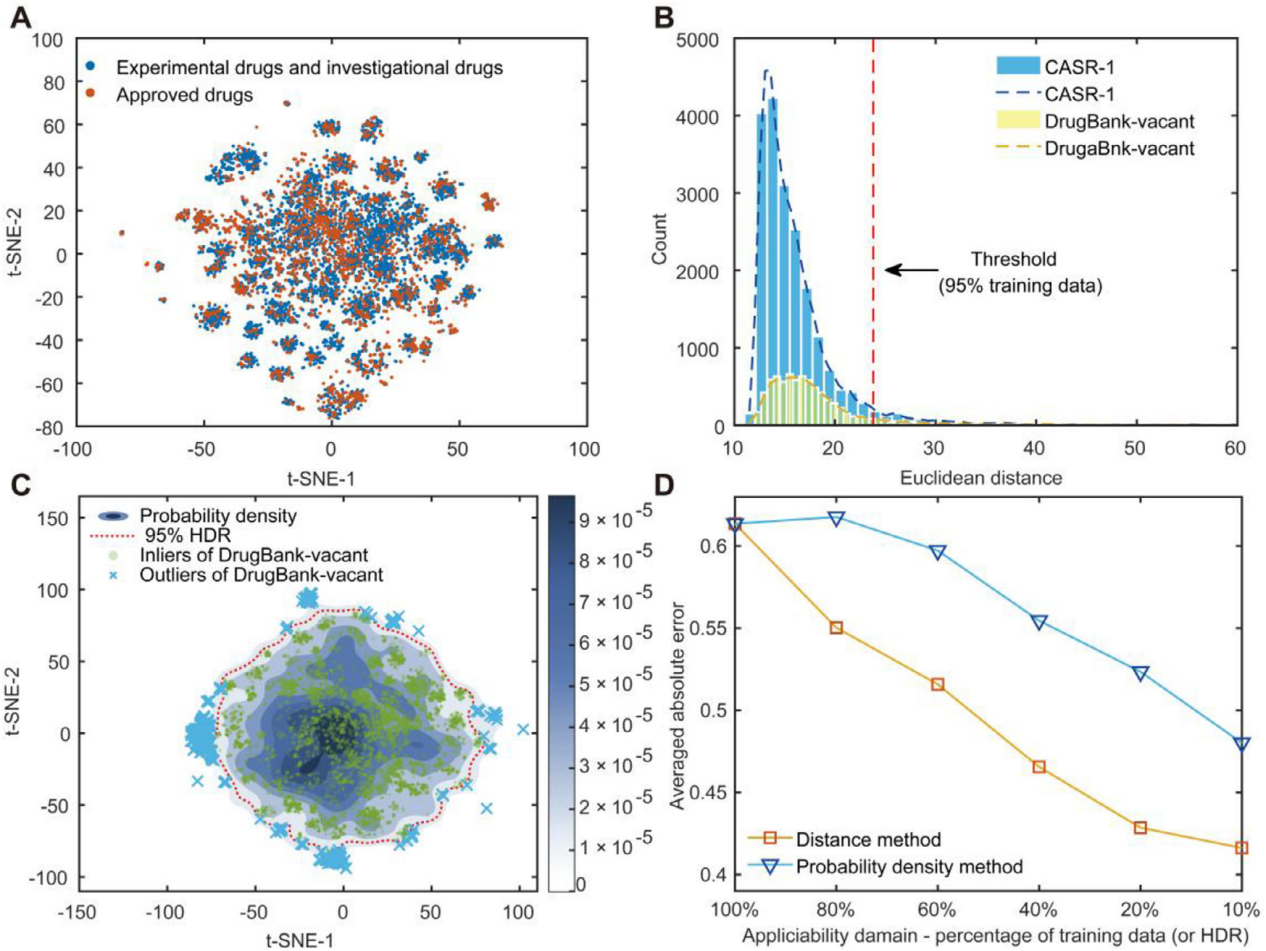
Applicability domain analysis. A) Chemical space of approved drugs and potential drugs. B) Applicability domain defined by Euclidean distance method. C) Applicability domain defined by probability density method. D) Changes of prediction error (5‐fold internal cross‐validation) for Stacking‐Lasso with the shrinking of applicability domain.

Decades of research have shown that drug compounds tend to cluster in small, compact regions within the broader chemical space.^[^ ^42^ ^]^ These clusters correspond to groups of molecules with similar structural or physicochemical properties. As illustrated in the 2D descriptor space derived from the 159 standardized RDKit descriptors using t‐SNE^[^ ^73^ ^]^ dimensionality reduction, potential new drugs from the DrugBank database align closely with the already approved drugs, forming discernible clusters (**Figure** **5**A). This observed clustering suggests that future drugs or drug‐like compounds are unlikely to occupy novel regions outside these clusters within the chemical space. In this sense, constructing predictive models that perform well within these currently accessible clusters are of high relevance.

To define the AD of the models developed in this study, two complementary methods were employed: the Euclidean distance method and the probability density method.^[^ ^74^, ^75^ ^]^ The Euclidean distance method computes the similarity of a given molecule to the training set based on standardized RDKit descriptors.^[^ ^76^ ^]^ Molecules exhibiting Euclidean distances falling within the 95th percentile threshold (< 23.8) were classified as residing within the AD. Using this approach, 89.14% of molecules in the DrugBank‐vacant subset were identified as falling within the model's AD (Figure 5B). Additionally, the probability density method evaluates whether molecules fall within the high‐density regions of the training set's descriptor space. By applying this method to the t‐SNE‐reduced space, it was found that 88.50% of DrugBank‐vacant molecules lie within the 95% highest density region (HDR) of CASR‐1 (> 1.99×10^−5^) (Figure 5C). These findings confirm that the majority of DrugBank‐vacant falls within the AD of the developed models, suggesting robust generalization capabilities for predicting drug solubility.

Moreover, we altered the AD‐defining thresholds, upon which the size of training set was adjusted. A consistent increase in model accuracy was observed with decreasing dataset size, which confirms the effectiveness of both AD‐defining methods in determining reliable prediction boundaries (Figure 5D).

**Figure 6:**
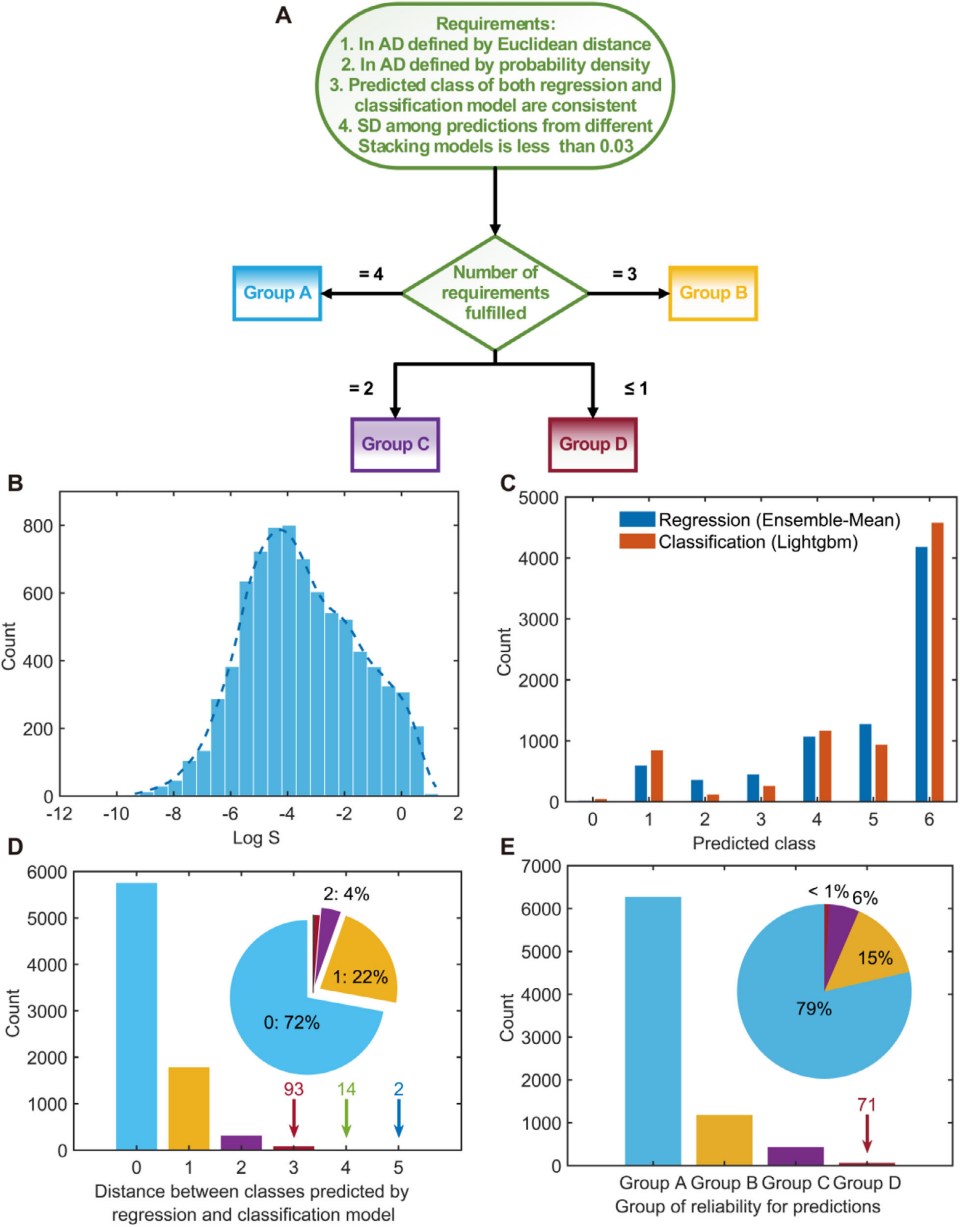
Data filling for DrugBank database. A) Overall workflow to define reliability of the predictions. B) Distribution of predicted Log S values from Stacking‐Lasso model. C) Comparison of the predicted solubility class from Stacking‐Lasso regression model, with the solubility class from LightGBM classification model. D) Difference between predicted class from regression model and classification model. E) Distribution of predicted values with different reliability groups.

Next, the solubility of DrugBank‐vacant molecules was predicted using the mean output from three Stacking models (Stacking‐Lasso, Stacking‐Ridge, and Stacking‐MLR), collectively referred to as Ensemble‐Mean. To assess the reliability of these predictions, all molecules were categorized into four groups based on the number of criteria they satisfied. Molecules meeting all four criteria were labeled as Group A (most reliable), while those satisfying one or none were labeled as Group D (least reliable), and those meeting three and two of the criteria were labeled Group B and C, respectively (**Figure** **6**A). The predicted solubility values exhibit a near‐normal distribution (Figure 6B), consistent with general expectations for solubility data. When these predictions were converted into solubility class, 72.34% match the results produced by the LightGBM classification model (Figure 6C). Moreover, 94% of the predictions fall within the nearest class to those made by LightGBM (Figure 6D), highlighting strong agreement between regression and classification approaches. Overall, 79% of DrugBank‐vacant molecules were classified as Group A and 15% as Group B, demonstrating the credibility of the populated predictions (Figure 6E).

These results underscore the utility of the AD framework in ensuring robust solubility predictions for unexplored drug‐like molecules. By leveraging the ‘wisdom of the crowd’ approach through ensemble modeling and systematically categorizing prediction reliability, this work provides a practical tool for populating missing solubility data in databases like DrugBank and guiding future drug development efforts.

### 2.7 Experimental Verification

To further evaluate the generalization ability of the models, experimental validation was conducted on several potential drugs in DrugBank‐vacant labeled as ‘Group A’ or ‘Group B’. The saturation shake‐flask method, a gold‐standard approach for solubility measurement, was employed for these experiments.^[^ ^43^, ^77^ ^]^ Compounds spanning a range of predicted solubility classes were selected to comprehensively assess model predictions, and as far as we know, they have no recorded experimental solubility in public chemical databases and literatures. Experimental validations were subsequently performed to evaluate the performance of our Stacking‐Lasso model (**Table** **4**). For the experimentally tested molecules, the Stacking‐Lasso model significantly outperforms other widely accepted tools and models (including ALOGPS, GSE, and Abraham solvation equation (ASE)^[^ ^78^ ^]^) on all metrics of RMSE, R^2^, %Log S ± 0.7, %Log S ± 1.0, and MAPD (Table S13, Supporting Information). Specifically, the RMSE between experimental and the Stacking‐Lasso predicted aqueous solubility is 0.436, closely aligning with the potential deviations of interlaboratory measurements (SD = 0.5 Log unit). Notably, the experimental solubility classes all match the predicted classes or fall within the adjacent categories, demonstrating strong agreement between predictions and actual values. These experimental results confirm the high reliability of our models for predicting the solubility of drug‐like compounds, particularly those within the defined AD.

**Table 4:** Results of solubility experiments.

Name	Predicted Log S [mol/L]	Predicted S [g/L]	Predicted Class^a^)	Predicted Class^b)^	Experimental Log S	Experimental Class	Absolute Error for Log S
Levodropropizine	−0.506	73.701	2	1	−0.839 ± 0.002	2	0.333
Deoxyinosine	−1.163	17.349	3	4	−1.392 ± 0.007	3	0.229
Edetate copper disodium	−0.094	320.643	1	1	0.310 ± 0.003	1	0.404
Thiamine disulfide	−1.379	23.499	3	1	−2.386 ± 0.055	4	1.007
4‐Iodo‐L‐phenylalanine	−2.270	1.563	4	5	−2.743 ± 0.167	5	0.473
4‐methylumbelliferyl β‐D‐glucoside	−1.919	4.076	4	3	−1.886 ± 0.016	4	0.033
7‐Deazaguanine	−2.240	0.863	5	4	−1.781 ± 0.068	4	0.449
Mdl‐29951	−3.764	0.052	6	6	−3.765 ± 0.166	6	0.023
Racecadotril	−3.785	0.063	6	6	−3.972 ± 0.146	6	0.206
Potassium hydrogen DL‐aspartate	0.682	823.936	1	1	0.784 ± 0.019	0	0.103

^a)^ From regression model (Ensemble‐Mean);

^b)^ From classification model (LightGBM).

## 3 Conclusion

This study presents a significant advancement in predicting aqueous solubility for drug and drug‐like molecules by compiling the largest aqueous solubility dataset to date and dual‐perspective modeling. The developed models demonstrate exceptional reliability and generalization capabilities across the chemical space of potential drugs, providing a valuable tool for assisting drug discovery. By integrating ML models with robust AD analysis, the solubility predictions for unrecorded entries in the DrugBank database were not only populated but also categorized into distinct reliability groups. These predictions serve as a critical resource for researchers and pharmaceutical developers. Experimental validation on ten compounds, selected from the DrugBank‐vacant entries, confirmed the high accuracy of the models, with predictions aligning closely with measured values. This marks the first time that solubility measurements are conducted for these specific compounds, further showcasing the practical applicability of the models.

Despite the promising results, DL models underperform the ensembles of statistical ML algorithms, given the current data limitations (particularly for compounds with extremely high or low solubility). The models have likely approached the limits of prediction accuracy and application scope based on the current dataset. However, the true potential of DL models remains untapped, as their ability to extract complex molecular features could yield even better predictions with access to larger, higher‐quality datasets.

To advance this field, it is crucial for researchers and organizations to share measured solubility data for unrecorded compounds, particularly those with unique and complex structures. Such efforts, coupled with detailed experimental methodologies, would enable the creation of more comprehensive datasets, supporting the development of even more advanced predictive models.

By bridging computational predictions with experimental validation, this work establishes a strong foundation for utilizing ML in practical drug development workflows, ultimately accelerating the discovery of new medical compounds.

## 4 Experimental Section

### DrugBank Database Curation

DrugBank (www.drugbank.ca) is a web‐enabled database containing comprehensive molecular information about drugs, including mechanisms, interactions, and targets.^[^ ^24^ ^]^ The data labeled as ‘small molecule’, ‘approved’, ‘experimental’, and ‘investigational’ were extracted from the DrugBank database, which encompasses drugs approved for commercialization by FDA and potential drug molecules under clinical or laboratory trials. For experimental solubility records in the DrugBank database, the following entries were excluded: 1) Entries with unspecified data source, temperature, solvent, and units of solubility, 2) Entries with ambiguous solubility descriptions that cannot be converted into solubility classes (non‑pharmacopoeial or overly wide solubility range, Table S14, Supporting Information), 3) Entries with temperatures outside the range of 25 ± 2 °C, and 4) Entries using solvents other than water. After the curation, the size of the DrugBank test set reduced from 1448 to 482.

The remaining entries were then cross‐checked with their respective data sources to ensure consistency. Entries that are inconsistent with their data sources were also discarded (e.g., mistaking the salt form of a compound for the compound itself). The resulting entries were divided into DrugBank set and DrugBank‐class set. The former is composed of solubility values and the latter is from entries with only solubility class. Salts and molecules containing metals were kept in this test set to evaluate models’ capability of solubility prediction for all kinds of drug‐like compounds. For the remaining entries in DrugBank database, the duplications with CASR‐1, CASR‐2, and external sets were excluded. The resulting dataset is termed DrugBank‐vacant.

### Classification of Solubility

The European Pharmacopoeia^[^ ^79^ ^]^ categorizes drugs into seven solubility classes: “very soluble”, “freely soluble”, “soluble”, “sparingly soluble”, “slightly soluble”, “very slightly soluble”, “practically insoluble or insoluble.” These categories correspond to the amount of water required to dissolve 1 g of drug: < 1, 1–10, 10–30, 30–100, 100–1000, 1000–10000, and > 10000 mL, respectively (Table S15, Supporting Information). All solubility values were converted to solubility classes according to this standard for classification tasks. Entries in the DrugBank database that provide only a solubility range (e.g., less than 0.01 g L^−1^) were also converted to solubility classes.

### COSMO‐RS Calculations

For molecules not included in the COSMObase 2022, COSMO files were generated using DFT calculations. First, 3D coordinates were obtained from PubChem and the geometry was optimized at the B3LYP/6‐31+G (d, p) using Gaussian 09.^[^ ^80^, ^81^ ^]^ Then, the optimized structures were converted to COSMO files in Gaussian at BP‐TZVP level. Optimization failed for 31 molecules, mostly due to extreme structural complexity or the presence of rare elements, and these compounds were discarded (Table S16, Supporting Information).

After preparing the input files, solubility was calculated using the COSMOthermX (version 2022). The required fusion energy was estimated by a QSPR approach with the default parameters in the COSMOthermX.^[^ ^16^ ^]^ Solubility calculations were conducted using the iterative method, which was found to be more accurate than the non‐iterative and SLE methods. Additionally, a correction of −2.5 kcal mol^−1^ per secondary or tertiary aliphatic amino group in the solute was applied due to a known systematic deviation of COSMO‐RS for secondary/tertiary aliphatic amines in protic solvents (e.g., water), which has already been suggested in the original literature of COSMO‐RS.^[^ ^17^, ^82^ ^]^

### Data Re‐Sampling Techniques

Data imbalance significantly impacts model performance in classification tasks. To address this concern, data re‐sampling techniques were employed to equalize the number of data points across classes and enhance sensitivity to minority classes. Three approaches were utilized: over‐sampling minority classes, under‐sampling majority classes, and combining both methods. Random Under‐sampling and Random Over‐sampling were implemented as basic methods.^[^ ^70^, ^71^ ^]^ The former randomly selects a subset of the majority class, while the latter randomly duplicates samples from the minority class. The Synthetic Minority Over‐sampling Technique^[^ ^68^ ^]^ (SMOTE) generates data points based on feature space interpolation. SMOTE selects a point *x*_i_ in the feature space of an original dataset and chooses *n* samples (*x*_i1_, *x*_i2_, …, *x*_in_) from its k‐nearest neighbors, generating new points along the lines between *x*_i_ and these neighbors. Variants of SMOTE include Borderline 1 and 2^[^ ^69^ ^]^ and SMOTEENN. The former is over‐sampling technique for identifying edge samples and the latter combines over‐sampling and under‐sampling to refine the balance between classes.^[^ ^72^ ^]^

### Machine Learning Environments

All SMILES strings provided by databases were converted to normalized canonical forms using the Python RDKit package to ensure uniform representation and simplify the identification of duplicate molecules. RDKit was also used to generate molecular descriptors. Statistical ML algorithms and stacking models were implemented using the python scikit‐learn^[^ ^83^ ^]^ library, which was also used for hyperparameter optimization. DL structures were built using PyTorch, with hyperparameter optimization performed via Optuna.^[^ ^84^ ^]^ Data standardization and metrics were processed using scikit‐learn, while data re‐sampling algorithms were applied using the imbalanced‐learn library.^[^ ^85^ ^]^ SHAP analyses were conducted using SHAP package.^[^ ^86^ ^]^

### Solubility Measurement

Molecules for solubility tests are shown in Figure S22 (Supporting Information), and the principles for these options are detailed in Supporting Information (Page 52). To avoid oxidization, most experimental procedures were conducted in an inert atmosphere in a glove box (Mikrouna MKUS2‐2309‐0069) that can control the water and oxygen concentration below 0.01 ppm. Excess solid drug was added to a vial to prepare supersaturated aqueous solutions. The vial was placed in a constant temperature mixer (Titan Technology, SD2‐100) at 25 °C and shaken for 48 h at a speed of 500 rpm. To ensure solid‐liquid equilibrium, solubility values were also measured after 72 h of mixing. As no differences were observed between 48‐ and 72‐h measurements, the solubility values were confirmed to be thermodynamic rather than kinetic. After equilibration, the solution was centrifugated at room temperature at 8000 rpm, and the supernatant was collected. The concentration of the supernatant was determined using the gravimetric method. A measured volume of the supernatant (e.g. 1 mL) was transferred to a pre‐weighted empty vial, dried in a vacuum oven at 70 °C, and re‐weighed. The difference between the initial and final weights represents the drug content in the saturated solution. Each compound was tested in triplicate to ensure accuracy. For an extremely insoluble compound named riboflavin tetrabutyrate (Figure S23, Supporting Information), whose solubility is hard to measure through gravimetric method or High‐Performance Liquid Chromatography (HPLC), ^1^H Nuclear Magnetic Resonance Spectra (^1^H NMR) is conducted on the saturated aqueous solution using a Bruker Advance III instrument. The result shows that no existence of solute in the solution, and thus the prediction of our model is relatively accurate (Figure S24, Supporting Information).

## Conflict of Interest

The authors declare no conflict of interest.

## Supporting information



Supporting Information

Supporting Information

## Data Availability

All data are available in the main text or the supplementary materials. Data involved in this work are provided as a separate excel file. Codes for model construction are available at: https://github.com/Thespidey1/Machine‐learns‐aqueous‐solubility‐of‐drugs.
