# Supplementary material for: Rethinking the AI Paradigm for Solubility Prediction of Drug‑Like Compounds with Dual‐Perspective Modeling and Experimental Validation
Source: Adv Sci (Weinh). 2025 Sep 25;12(46):e11667. doi: 10.1002/advs.202511667 (PMC12697872; doi:10.1002/advs.202511667)
Supplement: Supplementary file 1 — Supporting Information [file ADVS-12-e11667-s001.docx]

**Rethinking the AI Paradigm for Solubility Prediction of Drug‑like Compounds with Dual-Perspective Modeling and Experimental Validation**

*Supplementary Information*

Qilin Zhu^1,^ ^†^, Yuxin Qiu^1,^ ^†^, Guzhong Chen^2,^ *, Wenyao Chen^1^, Xiang Zhang^1^, Zhiwen Qi^1^, Xuezhi Duan^1^, De Chen^3^, Zhen Song^1,^ *

*^1^State Key Laboratory of Chemical Engineering, School of Chemical Engineering, East China University of Science and Technology, 130 Meilong Road, Shanghai 200237, China*

*^2^Department of Chemical Engineering, Columbia University, New York, NY 10027*

*^3^Department of Chemical Engineering, Norwegian University of Science and Technology, Trondheim, Norway*

*^†^ These two authors contributed equally to this work.*

**Corresponding authors: gc3115@columbia.edu; songz@ecust.edu.cn*

**This file includes:**

Supplementary Text

Figure S1 to S24

Table S1 to S16

**Contents**

[Supplementary Note 1: Literature review 3](#_Toc207462322)

[Supplementary Note 2: More datasets information 4](#_Toc207462323)

[Supplementary Note 3: Evaluation metrics 8](#_Toc207462324)

[Supplementary Note 4: Feature selection 10](#_Toc207462325)

[Supplementary Note 5: Hyperparameter optimization for regression tasks 13](#_Toc207462326)

[Supplementary Note 6: Detailed regression results 14](#_Toc207462327)

[Supplementary Note 7: Correlation between predictions by different models and the SD among the Stacking models’ predictions 26](#_Toc207462328)

[Supplementary Note 8: Dotted SHAP figures 28](#_Toc207462329)

[Supplementary Note 9: Hyperparameter optimization for classification tasks 30](#_Toc207462330)

[Supplementary Note 10: Detailed classification results 31](#_Toc207462331)

[Supplementary Note 11: Detailed results of applying data re-sampling techniques 38](#_Toc207462332)

[Supplementary Note 12: Detailed results of applying data re-sampling technique 40](#_Toc207462333)

[Supplementary Note 13: Examples of discarded ambiguous entries of DrugBank database 41](#_Toc207462334)

[Supplementary Note 14: Conversion of solubility values and the corresponding classes 42](#_Toc207462335)

[Supplementary Note 15: Dimension reduction 43](#_Toc207462336)

[Supplementary Note 16: Applicability domain analysis 44](#_Toc207462337)

[Supplementary Note 17: General solubility equation and Abraham solubility equation 45](#_Toc207462338)

[Supplementary Note 18: COSMO-RS calculations 46](#_Toc207462339)

[Supplementary Note 19: Molecules for experiments 49](#_Toc207462340)

[Supplementary Note 20: An extremely insoluble (~ $\boldsymbol{1} \times\boldsymbol{10}-\boldsymbol{6} \mathbf{g}/\mathbf{L}$) molecule in experiments 50](#_Toc207462341)

[Supplementary Note 21: Principles for the selection of experimentally tested compounds 52](#_Toc207462342)

[References: 53](#_Toc207462343)

# Supplementary Note 1: Literature review

**Table S1. Summary of literature review of works in recent 5 years.**

| **Reference** | **R^2^** | **RMSE** | **Model validation** | **Number of data points** | **Method** |
| --- | --- | --- | --- | --- | --- |
| This work | 0.823 | 0.962 | Cross validation, Test set | 19942 | Stacking |
| Ghanavati et al.^[1]^ | 0.918 | 0.613 | Test set | 3942 | XGB |
| Tayyebi et al.^[2]^ | 0.880 | 0.640 | Test set | > 8438 | RF |
| Zhu et al.^[3]^ | 0.755 | 0.959 | Test set | > 5700 | XGB |
| Lowe et al.^[4]^ | 0.820 | 0.970 | Cross validation | 8037 | RF |
| Avdeef^[5]^ | 0.660-0.830 | 0.750-1.050 | Cross validation, Test set | 6355 | RF |
| Tosca et al.^[6]^ | 0.510 | 1.370 | Cross validation, Test set | 270 | ANN |
| Ramos et al.^[7]^ | / | 0.983-1.205 | Test set | 9982 | Deep ensemble LSTM |
| Chen et al.^[8]^ | 0.757 ~ 0.914 | 0.687 ~ 1.079 | Test set | 1144 ~ 9943 | TAGCN |
| Panapitiya et al.^[9]^ | 0.768 | 1.084 | Cross validation, Test set | 17149 | MDM |
| Cui et al.^[10]^ | 0.720 ~ 0.790 | 0.988 ~ 1.151 | Cross validation, Test set | 9943 | Resnet |
| Francoeur et al.^[11]^ | / | 1.459 | Cross validation, Test set | 9982 | Transformer |

# Supplementary Note 2: More datasets information

**
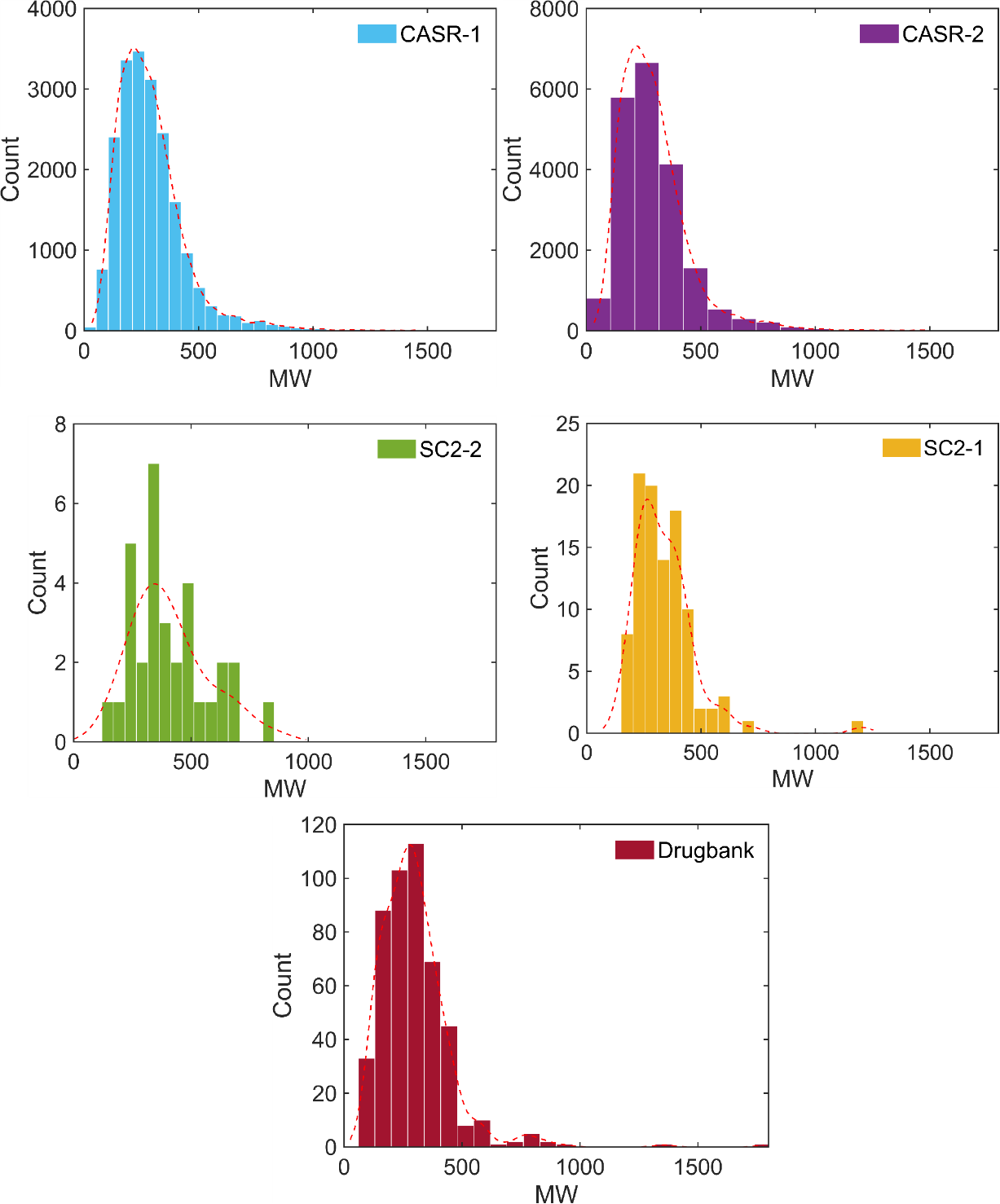
**

**Figure S1. Molecular weight distributions of datasets in this work.**

**
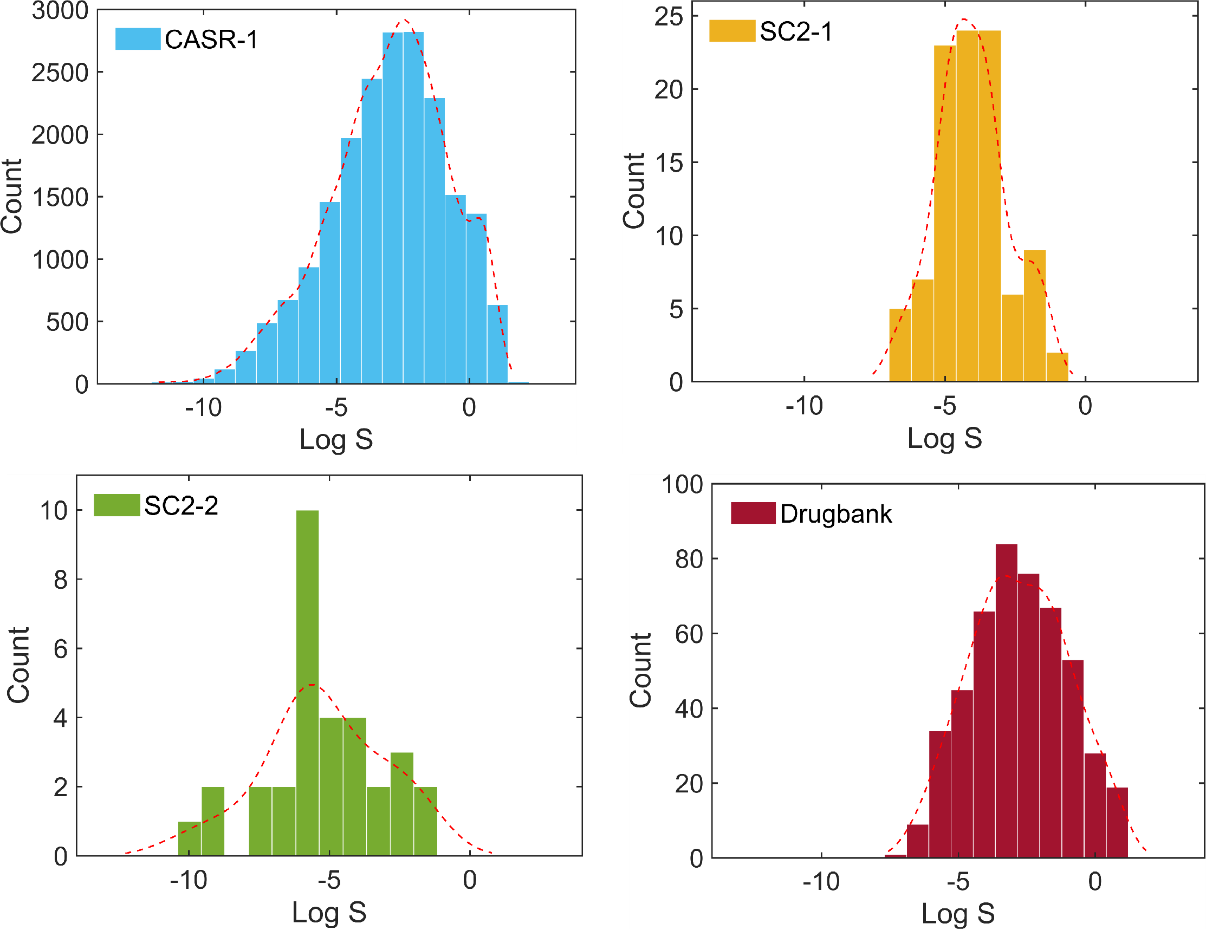
**

**Figure S2. Log S distributions of datasets in this work.**

**
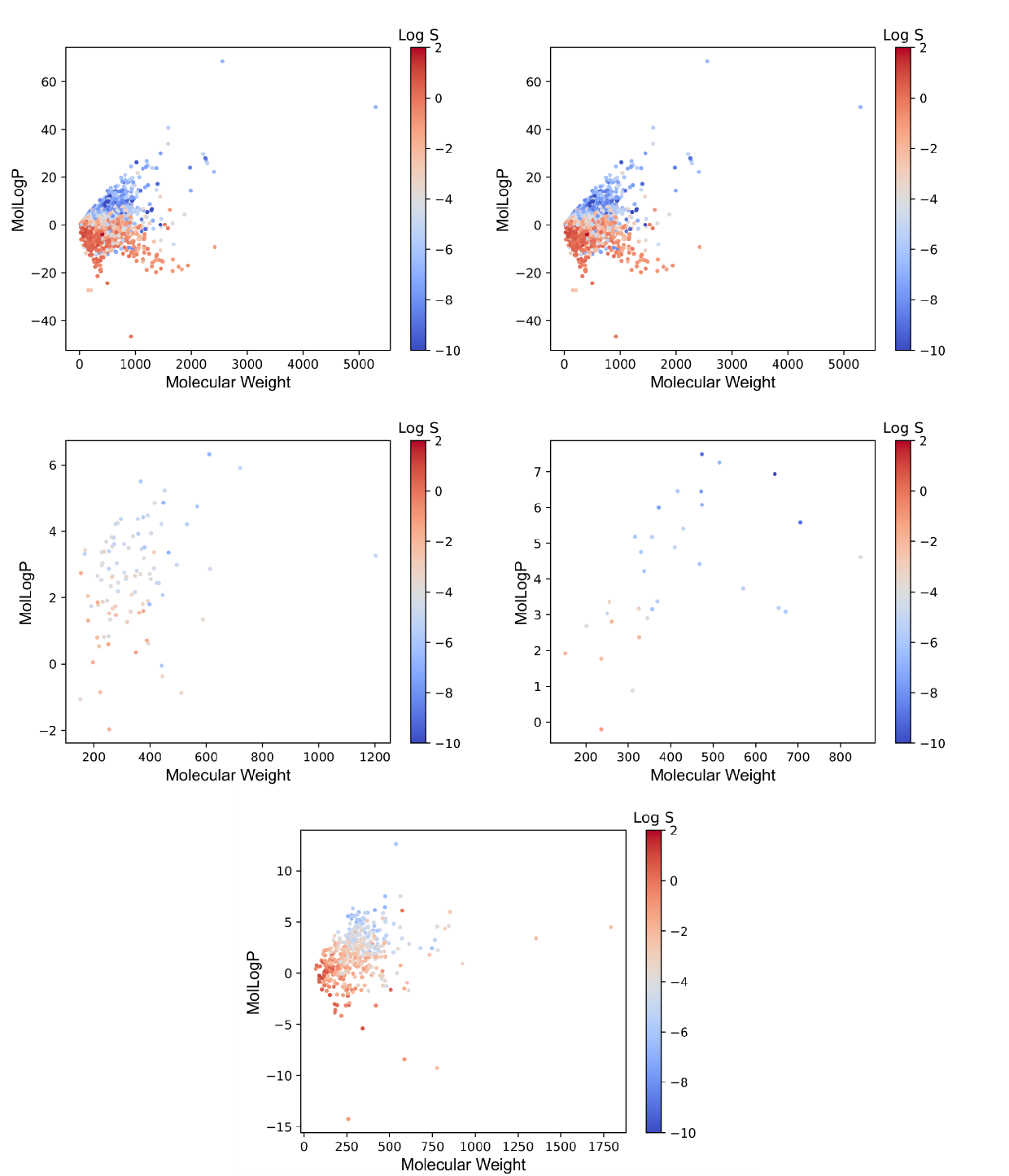
**

**Figure S3. Chemical space of the datasets used in this study, defined by molecular weight (MW), Log P, and Log S.** From left to right and top to bottom, the datasets are CASR-1, CASR-2, SC2-1, SC2-2, and DrugBank, respectively. The data points exhibit similar distributions across all datasets. The training sets span a significantly larger chemical space compared to the external test sets.

**Standard SMILES strings**

All SMILES are first checked and invalid ones are marked. Valid SMILES are then ‘normalized’ to make sure a fixed way of writing the SMILES string. Finally, SMILES are ‘standardized’ to canonical SMILES. The resulting SMILES are referred to as ‘Standard SMILES’. Code to achieve this procedure is listed below:

# mol = Chem.MolFromSmiles(datastr)
# mol.UpdatePropertyCache(strict=False)
# Chem.SanitizeMol(mol, Chem.SANITIZE_SYMMRINGS | Chem.SANITIZE_SETCONJUGATION | Chem.SANITIZE_SETHYBRIDIZATION)
# normalizer = rdMolStandardize.Normalizer()
# mol = normalizer.normalize(mol)
# smi = Chem.MolToSmiles(mol, isomericSmiles=True, canonical=True)
# stand_smiles = rdMolStandardize.StandardizeSmiles(smi)

# Supplementary Note 3: Evaluation metrics

**Regression tasks**

In regression tasks, three evaluation metrics—coefficient of determination (R^2^), root mean squared error (RMSE), and mean absolute percentage deviation (MAPD)—are utilized, along with two specific metrics designed for solubility prediction: %Log S ± 0.7 and %Log S ± 1.0. These metrics are defined as follows:

$$\begin{aligned} R^{2}=1- \frac{\sum_{i} \left( \hat{y}_{i} - y_{i} \right)^{2}}{\sum_{i} \left( \bar{y} - y_{i} \right)^{2}}\#\left( 1 \right) \end{aligned}$$

$$\begin{aligned} RMSE=\sqrt{\frac{1}{m} \sum_{i=1}^{m} \left( \hat{y}_{i} - y_{i} \right)^{2}}\#\left( 2 \right) \end{aligned}$$

$$\begin{aligned} MAPD=\frac{1}{m}\sum_{i=1}^{m} \left| \frac{\hat{y}_{i} - y_{i}}{y_{i}} \right|\times100\%\#\left( 3 \right) \end{aligned}$$

where $y_{i}$ represents the true value, $\hat{y}_{i}$ denotes the predicted value, $\hat{y}_{i}$ is the mean of all true values, and m is the total number of data points. %Log S ± 0.7 and %Log S ± 1.0 indicate the percentage of predictions within ± 0.7 and ± 1.0 Log units of the true solubility, respectively.

**Classification tasks**

For classification tasks, six widely used metrics are employed to provide a comprehensive evaluation of predictions for both majority and minority classes. Each class is treated as the positive class while considering all others as negative. The metrics are defined as follows:

$$\begin{aligned} Accuracy=\frac{TP+TN}{TP+FP+TN+FN}\#\left( 4 \right) \end{aligned}$$

$$\begin{aligned} Precision =\frac{TP}{TP+FP}\#\left( 5 \right) \end{aligned}$$

$$\begin{aligned} Recall =\frac{TP}{TP+FN}\#\left( 6 \right) \end{aligned}$$

$$\begin{aligned} F1 = 2 Precision\times\frac{Recall}{Precision+Recall}\#\left( 7 \right) \end{aligned}$$

Here, TP, FP, TN, and FN represent the number of true positives, false positives, true negatives, and false negatives, respectively.

The receiver operating characteristic curve (ROC curve) illustrates the trade-off between the true positive rate and the false positive rate, while the precision-recall curve (PR curve) shows the relationship between precision and recall as the confidence threshold varies. The area under the ROC curve (AUC) and the area under the PR curve (AP) are used as summary metrics for these curves.

After computing these metrics for each class, the final model performance is evaluated using the macro-average method, which calculates the average values of the metrics across all classes.

# Supplementary Note 4: Feature selection

**Table S2. Removed descriptors because of calculation failure for some molecules.**

| **Feature index** | **Feature name** |
| --- | --- |
| 1 | MaxPartialCharge |
| 2 | MinPartialCharge |
| 3 | MaxAbsPartialCharge |
| 4 | MinAbsPartialCharge |
| 5 | BCUT2D_MWHI |
| 6 | BCUT2D_MWLOW |
| 7 | BCUT2D_CHGHI |
| 8 | BCUT2D_CHGLO |
| 9 | BCUT2D_LOGPHI |
| 10 | BCUT2D_LOGPLOW |
| 11 | BCUT2D_MRHI |
| 12 | BCUT2D_MRLOW |

**Table S3. Feature selection for regression tasks by feature importance^a)^.**

| **Selection criterion** | All descriptors | > 0.0001^b^ | > 0.0003 | > 0.001 | > 0.003 | > 0.005 | Top 10 |
| --- | --- | --- | --- | --- | --- | --- | --- |
| **Number of descriptors** | 159 | 129 | 114 | 77 | 48 | 26 | 10 |
| **RMSE** | 1.036 | 1.042 | 1.043 | 1.047 | 1.059 | 1.066 | 1.125 |
| **R^2^** | 0.794 | 0.792 | 0.791 | 0.790 | 0.785 | 0.782 | 0.757 |

^a)^Total results for 5-fold cross-validation; ^b)^‘>0.0001’ means keeping all descriptors with feature importance > 0.0001, and so forth.

**Table S4. Feature selection for classification tasks by feature importance^a)^.**

| **Selection criterion** | All descriptors | > 0.0001^a^ | > 0.0003 | > 0.001 | > 0.003 | > 0.01 | Top 10 |
| --- | --- | --- | --- | --- | --- | --- | --- |
| **Number of descriptors** | 159 | 147 | 135 | 104 | 71 | 42 | 10 |
| **Accuracy** | 0.652 | 0.651 | 0.653 | 0.652 | 0.652 | 0.652 | 0.623 |
| **Precision** | 0.544 | 0.538 | 0.538 | 0.538 | 0.542 | 0.546 | 0.505 |
| **Recall** | 0.471 | 0.467 | 0.469 | 0.469 | 0.471 | 0.471 | 0.442 |
| **F1** | 0.486 | 0.481 | 0.483 | 0.482 | 0.486 | 0.487 | 0.455 |
| **AUC** | 0.894 | 0.894 | 0.894 | 0.894 | 0.894 | 0.893 | 0.873 |
| **AP** | 0.539 | 0.536 | 0.535 | 0.537 | 0.540 | 0.533 | 0.495 |

^a)^Total results for 5-fold cross-validation.

# Supplementary Note 5: Hyperparameter optimization for regression tasks

A grid search method is applied with 5-fold cross-validation on the CASR-1 dataset to identify the optimal hyperparameters for all algorithms. Stacking models are constructed using base learners with their optimized hyperparameters. For stacking models, the hyperparameters of the meta-learners are also specified. The optimal hyperparameters are detailed below:

**Table S5. Optimized hyperparameters in regression.**

| **Model** | **Hyperparameter** | **Value** |
| --- | --- | --- |
| RF | n_estimators | 400 |
|  | max_depth | 50 |
| XGB | n_estimators | 500 |
|  | max_depth | 7 |
|  | learning_rate | 0.07 |
| LightGBM | n_estimators | 3000 |
|  | max_depth | 5 |
|  | learning_rate | 0.07 |
|  | num_leaves | 30 |
| SVR | kernel | ‘rbf’ |
|  | C | 10 |
|  | gamma | 0.004 |
| Stacking-MLR | / | / |
| Stacking-Ridge | fit_intercept | False |
|  | positive | True |
|  | alpha | 0.001 |
| Stacking-Lasso | fit_intercept | False |
|  | positive | True |
|  | alpha | 0.1 |

# Supplementary Note 6: Detailed regression results

The regression results are presented in the order of internal cross-validation (CV), SC2-1, SC2-2, and DrugBank, respectively, arranged from left to right and top to bottom. The same ordering is applied to the classification results in the subsequent section. For AutoML models, cross-validation results are not provided because evaluated by a different approach.

**Table S6. Regression performance on internal set by 5-fold cross-validation^a, b^.**

| **Method** | **R^2^** | **RMSE** | **%Log S ± 0.7** | **%Log S ± 1.0** | **MAPD** | **Regression score** |
| --- | --- | --- | --- | --- | --- | --- |
| RF | 0.792 | 1.042 | 66.3 | 78.4 | 190.1 | 0 |
| XGB | 0.811 | 0.994 | 69.2 | 80.6 | 128.0 | 0 |
| LightGBM | 0.810 | 0.996 | 69.7 | 80.8 | 151.6 | 0 |
| SVR | 0.798 | 1.026 | 69.5 | 80.0 | 145.3 | 0 |
| Stacking-MLR | **0.823** | **0.962** | 71.1 | 81.8 | 117.2 | 2 |
| Stacking-Ridge | 0.822 | 0.963 | 71.5 | **82.2** | 121.6 | 1 |
| Stacking-Lasso | **0.823** | **0.962** | **71.6** | 82.1 | **116.6** | **4** |
| Transformer-CNN | 0.773 | 0.986 | 70.3 | 81.1 | 121.2 | 0 |
| GNN | 0.792 | 1.043 | 65.5 | 78.8 | 121.0 | 0 |

^a^Total results for 5-fold cross-validation.

^b^Best value for each metric is in **bold**.

**Table S7. Regression performance on three external test sets^a), b)^.**

| **Database** | **Method** |  | | | **Metrics** | | | |
| --- | --- | --- | --- | --- | --- | --- | --- | --- |
|  |  | R^2^ | RMSE | %Log S ± 0.7 | | %Log S ± 1.0 | MAPD | Regression score |
| **SC2-1** | RF | 0.561 | 0.839 | 67.0 | | 80.0 | 15.2 | 0 |
|  | XGB | 0.572 | 0.829 | 65.0 | | 79.0 | 16.3 | 0 |
|  | LightGBM | 0.590 | 0.811 | **71.0** | | 80.0 | **15.1** | 2 |
|  | SVR | 0.528 | 0.870 | 62.0 | | 76.0 | 17.3 | 0 |
|  | Stacking-MLR | **0.610** | **0.791** | 68.0 | | **84.0** | 15.5 | **3** |
|  | Stacking-Ridge | **0.610** | **0.791** | 68.0 | | **84.0** | 15.5 | **3** |
|  | Stacking-Lasso | 0.600 | 0.801 | 68.0 | | 83.0 | 15.6 | 0 |
|  | AutoML | 0.607 | 0.794 | 63.0 | | 83.0 | 15.2 | 0 |
|  | Transformer-CNN | 0.571 | 0.829 | 64.0 | | 78.0 | 16.3 | 0 |
|  | GNN | 0.528 | 0.870 | 60.0 | | 80.0 | 19.4 | 0 |
| **SC2-2** | RF | 0.793 | 0.973 | 56.3 | | **78.1** | **7.9** | 2 |
|  | XGB | 0.758 | 1.053 | 53.1 | | 65.6 | 9.6 | 0 |
|  | LightGBM | 0.733 | 1.106 | 50.0 | | 68.8 | 10.6 | 0 |
|  | SVR | 0.729 | 1.116 | 53.1 | | 62.5 | 9.0 | 0 |
|  | Stacking-MLR | 0.779 | 1.006 | 56.3 | | 59.4 | 8.7 | 0 |
|  | Stacking-Ridge | 0.779 | 1.007 | 56.3 | | 59.4 | 8.7 | 0 |
|  | Stacking-Lasso | 0.769 | 1.029 | 56.3 | | 59.4 | 8.8 | 0 |
|  | AutoML | 0.773 | 1.020 | 46.9 | | 71.9 | 9.2 | 0 |
|  | Transformer-CNN | 0.718 | 1.137 | 56.2 | | 68.8 | 10.0 | 0 |
|  | GNN | **0.840** | **0.856** | **59.4** | | 65.6 | 9.3 | **3** |
| **DrugBank** | RF | 0.717 | 0.945 | 70.1 | | 81.1 | 191.7 | 0 |
|  | XGB | 0.734 | 0.917 | 69.3 | | 82.4 | 184.0 | 0 |
|  | LightGBM | 0.691 | 0.989 | 67.0 | | 80.5 | 174.5 | 0 |
|  | SVR | 0.718 | 0.945 | 69.5 | | 78.8 | 177.9 | 0 |
|  | Stacking-MLR | 0.735 | 0.914 | 71.2 | | 82.6 | 149.4 | 0 |
|  | Stacking-Ridge | 0.735 | 0.914 | 71.2 | | 82.6 | 149.2 | 0 |
|  | Stacking-Lasso | **0.739** | **0.909** | 71.4 | | 82.2 | **148.2** | **3** |
|  | AutoML | 0.715 | 0.949 | **72.2** | | **82.8** | 177.8 | 2 |
|  | Transformer-CNN | 0.698 | 0.977 | 68.7 | | 80.9 | 240.0 | 0 |
|  | GNN | 0.701 | 0.972 | 66.2 | | 78.0 | 202.6 | 0 |
| **All** | RF | 0.748 | 0.930 | 68.9 | | 80.8 | 153.4 | 0 |
|  | XGB | 0.759 | 0.911 | 67.8 | | 81.0 | 147.6 | 0 |
|  | LightGBM | 0.728 | 0.969 | 66.8 | | 79.8 | 134.0 | 0 |
|  | SVR | 0.742 | 0.943 | 67.4 | | 77.5 | 142.9 | 0 |
|  | Stacking-MLR | 0.764 | 0.900 | 69.9 | | 81.6 | 120.2 | 0 |
|  | Stacking-Ridge | 0.764 | 0.900 | 69.9 | | 81.6 | 120.1 | 0 |
|  | Stacking-Lasso | **0.766** | **0.899** | **70.1** | | 81.1 | **119.3** | **4** |
|  | AutoML | 0.749 | 0.929 | 69.4 | | **82.3** | 142.5 | 1 |
|  | Transformer-CNN | 0.730 | 0.964 | 67.3 | | 79.8 | 191.6 | 0 |
|  | GNN | 0.738 | 0.950 | 64.8 | | 77.7 | 162.7 | 0 |

^a)^Total results of five repetitions; ^b)^Best value for each metric is in **bold**.

**Table S8. Performance of GSE, ASE and Stacking-Lasso model on the external test sets.**

| **Database** | **Method** | **Metrics** | | | | | |
| --- | --- | --- | --- | --- | --- | --- | --- |
|  |  | R^2^ | RMSE | %Log S ± 0.7 | %Log S ± 1.0 | MAPD | Regression score |
| SC2-1 | GSE | 0.216 | 1.121 | 52.0 | 71.0 | 22.5 | 0 |
|  | ASE | -9.310 | 4.065 | 37.0 | 52.0 | 55.2 | 0 |
|  | Modified ASE | 0.024 | 1.251 | 47.0 | 67.0 | 22.2 | 0 |
|  | Stacking-Lasso | **0.610** | **0.801** | **68.0** | **83.0** | **15.6** | **5** |
| SC2-2 | GSE | 0.686 | 1.199 | 40.6 | 56.2 | 22.0 | 0 |
|  | ASE | -2.079 | 3.118 | 65.6 | 71.9 | 27.2 | 0 |
|  | Modified ASE | 0.445 | 1.245 | **68.8** | **75.0** | 12.8 | 2 |
|  | Stacking-Lasso | **0.769** | **1.029** | 56.3 | 59.4 | **8.8** | **3** |
| DrugBank | GSE | 0.209 | 1.579 | 46.5 | 62.8 | 168.5 | 0 |
|  | ASE | -10.148 | 5.936 | 40.5 | 49.5 | 385.9 | 0 |
|  | Modified ASE | 0.313 | 1.473 | 45.1 | 58.0 | 385.3 | 0 |
|  | Stacking-Lasso | **0.739** | **0.909** | **71.4** | **82.2** | **148.2** | **5** |


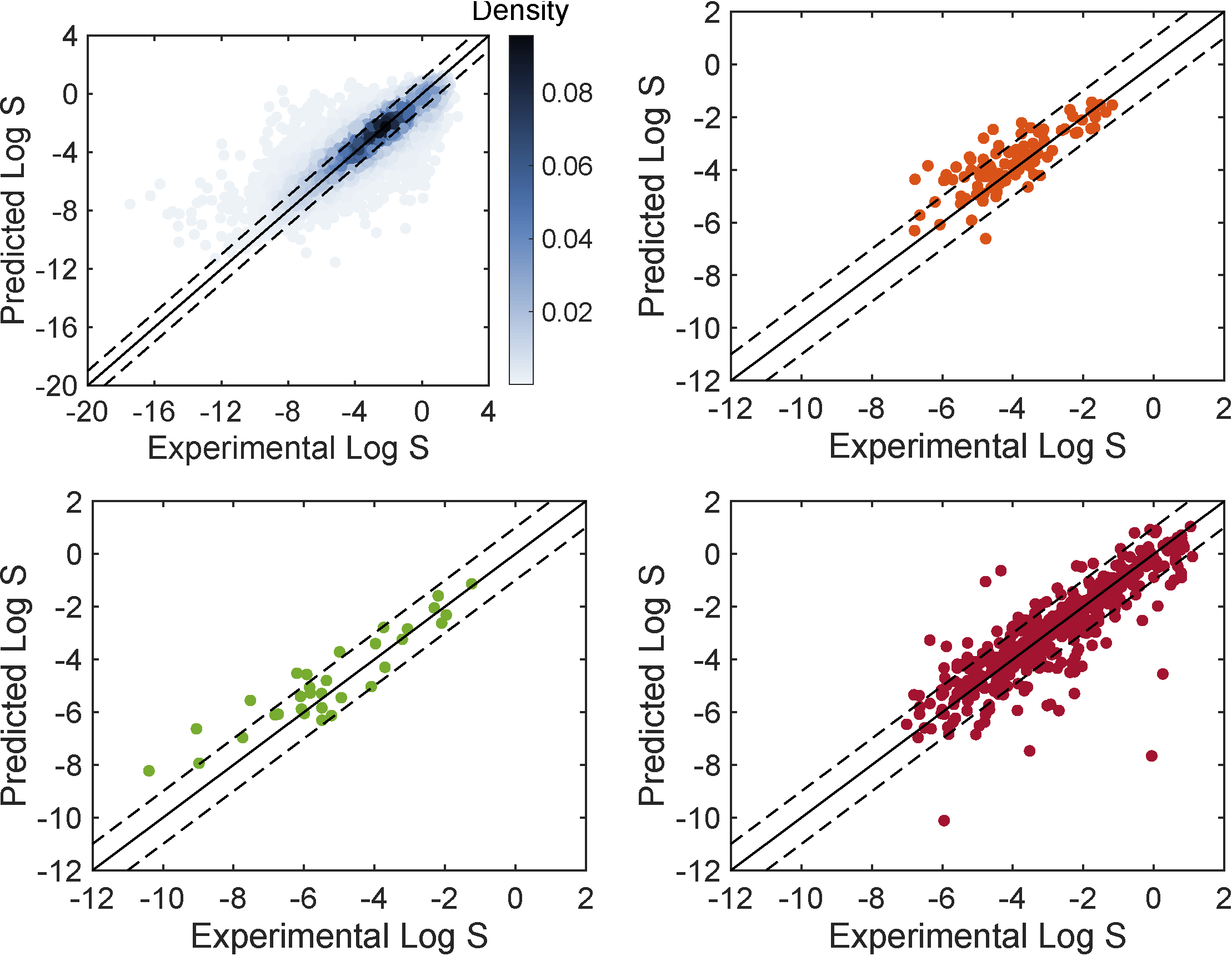


**Figure S4. Correlation plots for RF.**


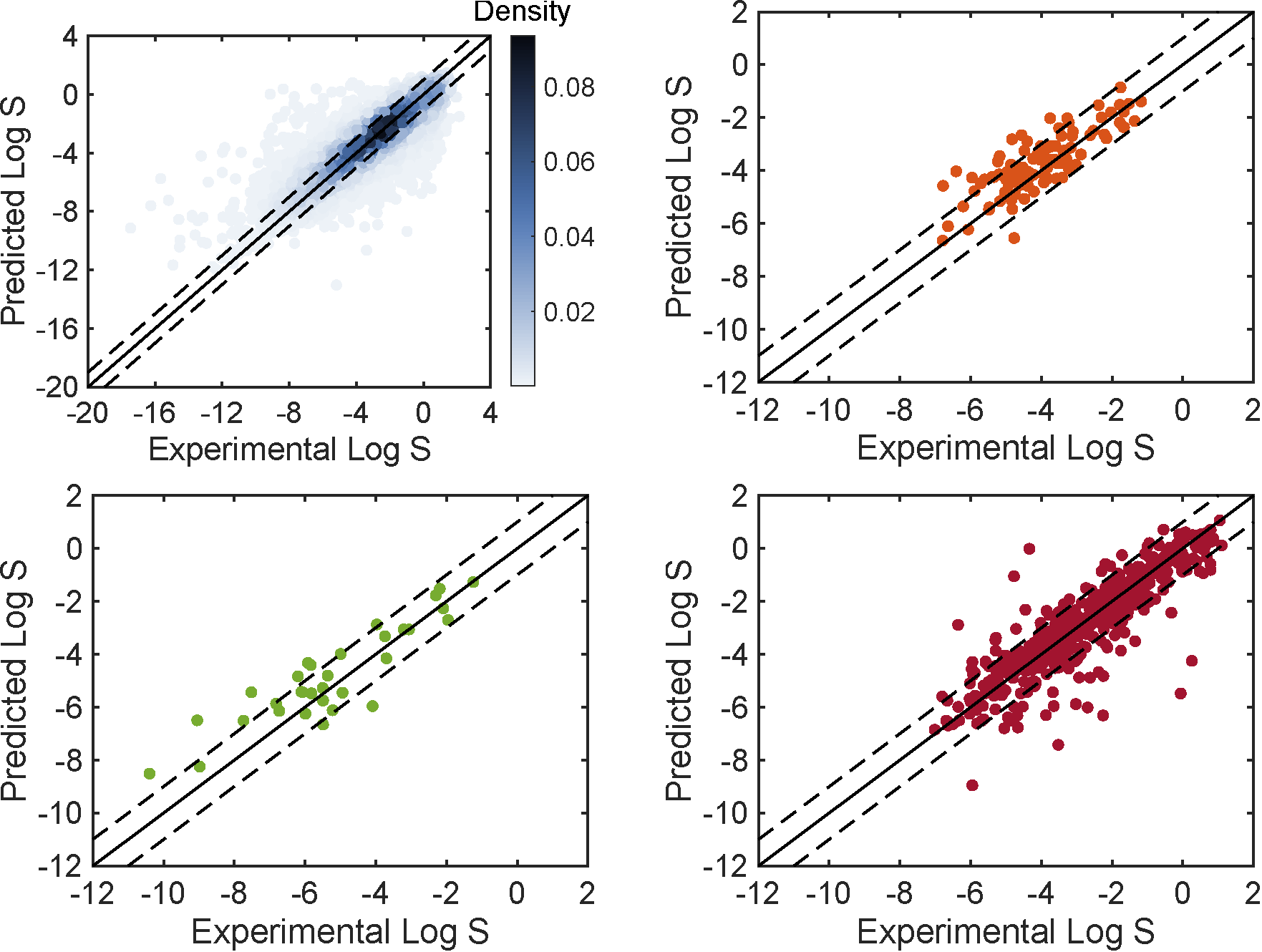


**Figure S5. Correlation plots for XGB.**


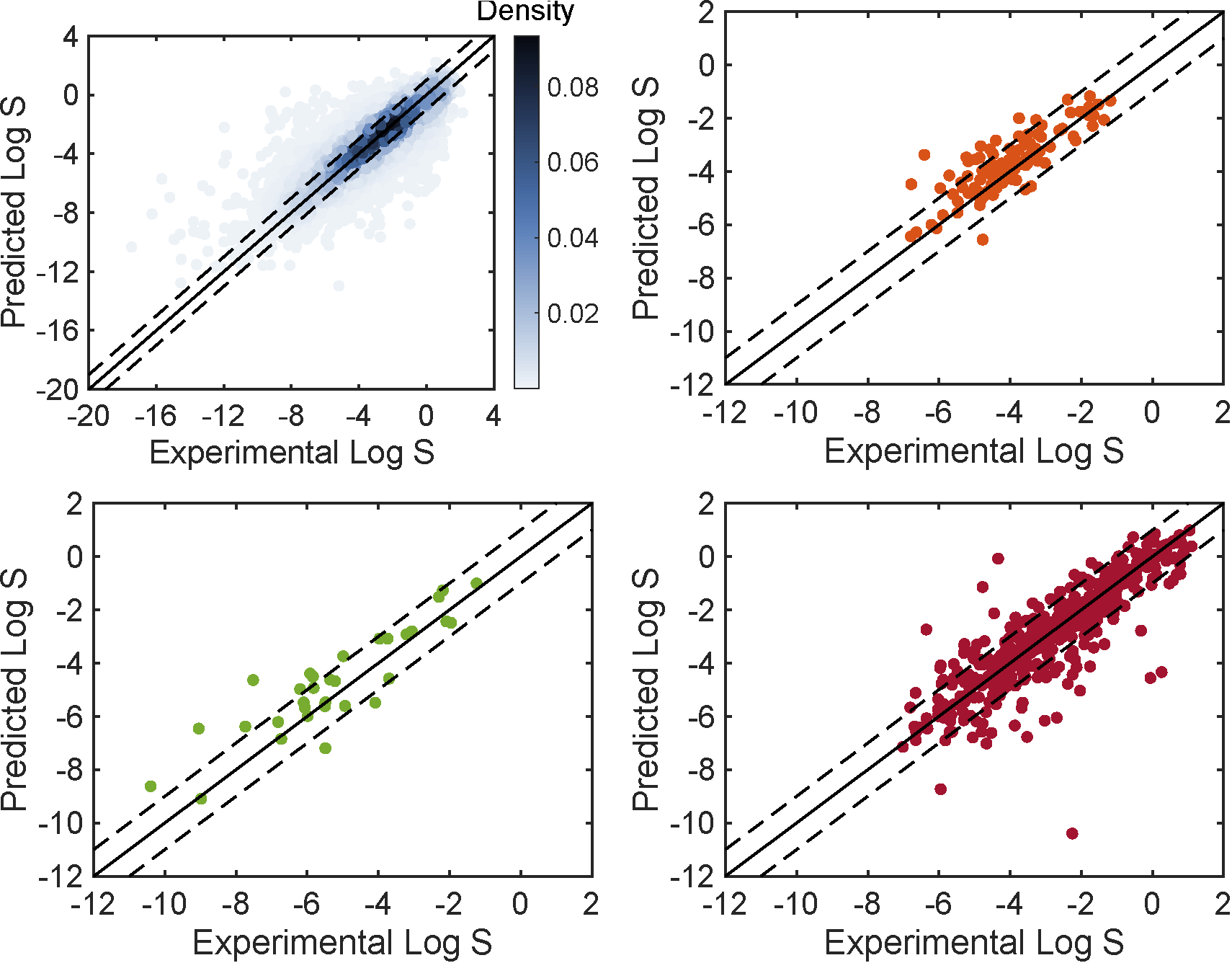


**Figure S6. Correlation plots for LightGBM.**


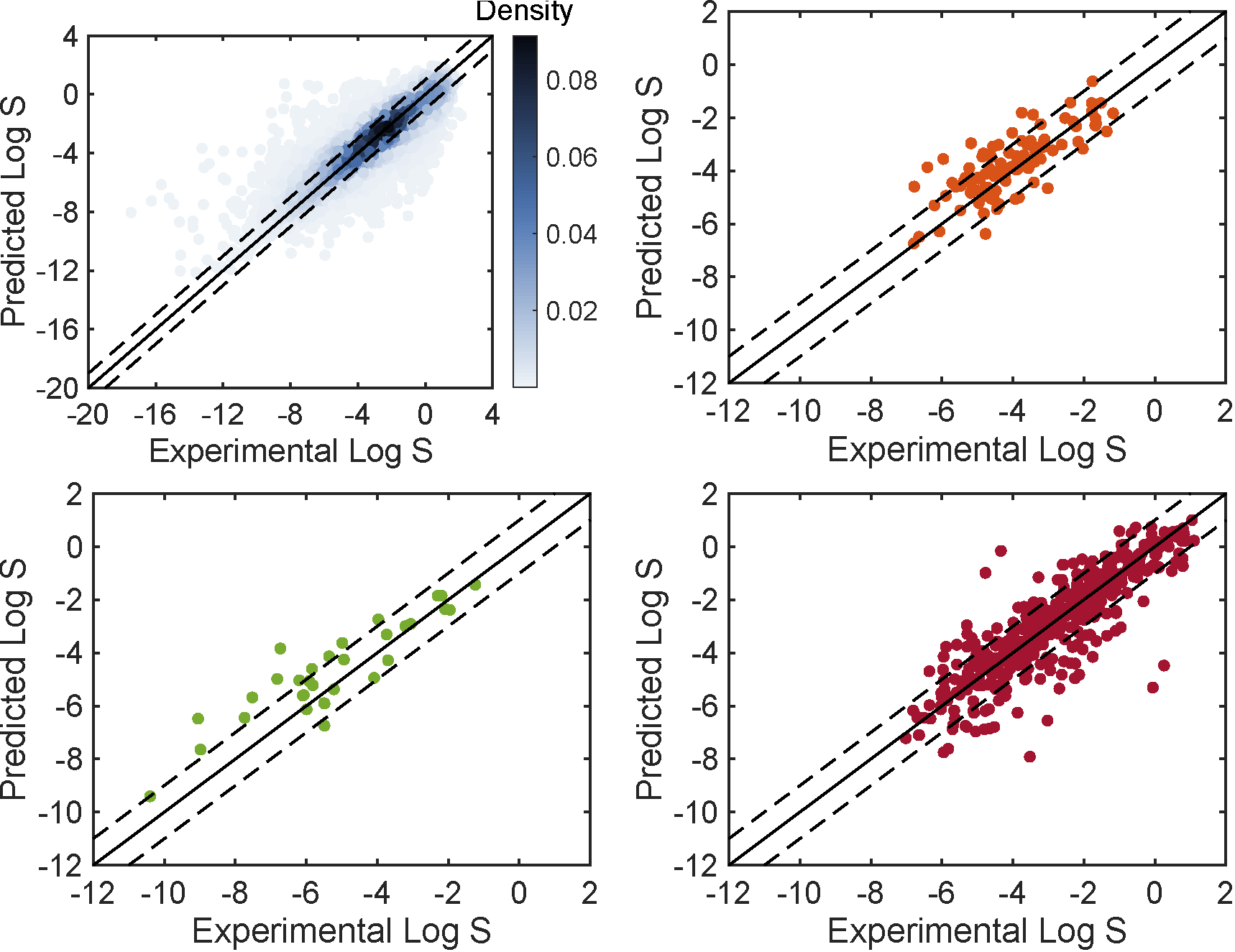


**Figure S7. Correlation plots for SVR.**


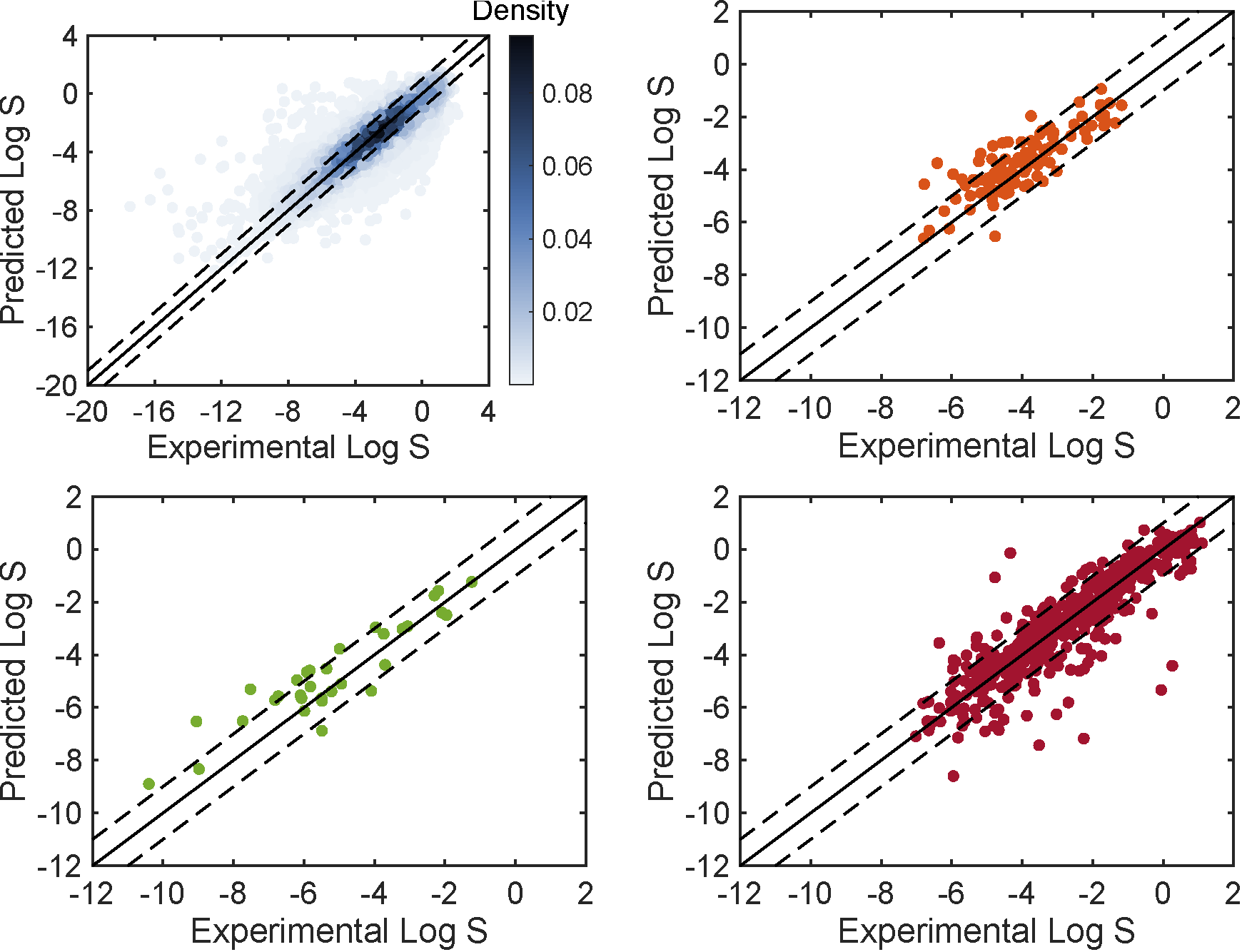


**Figure S8. Correlation plots for Stacking-MLR.**


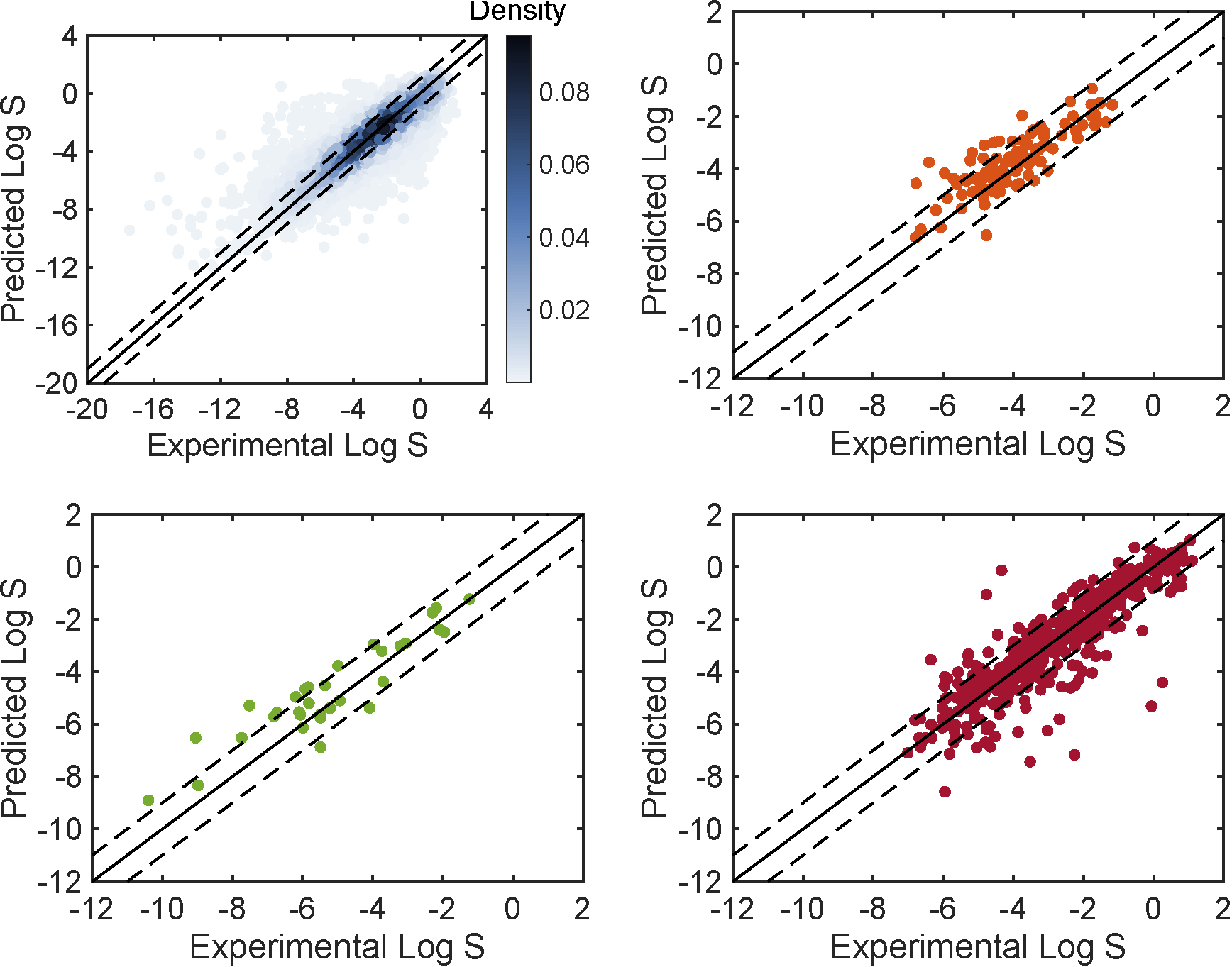


**Figure S9. Correlation plots for Stacking-Ridge.**


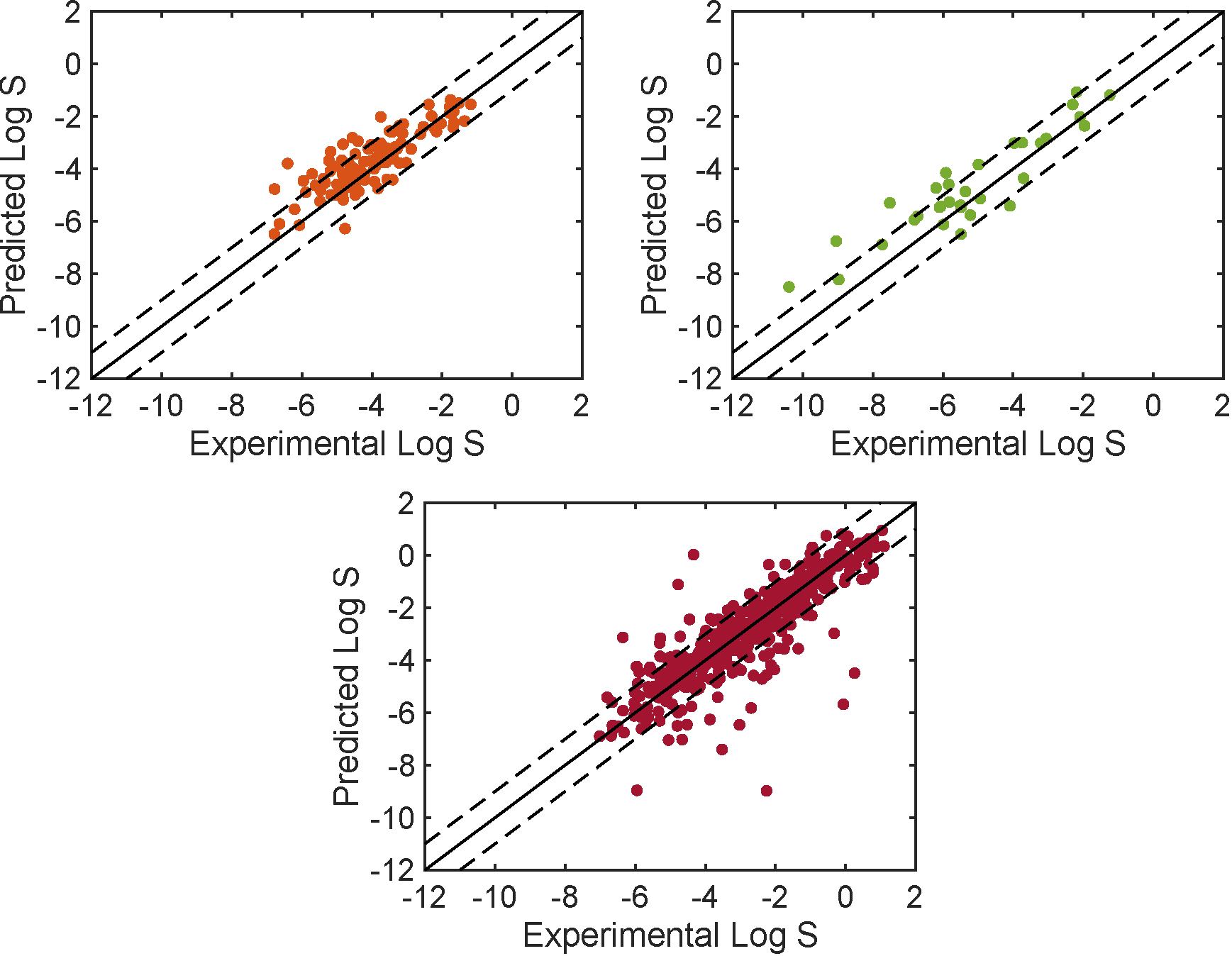


**Figure S10. Correlation plots for AutoML.**


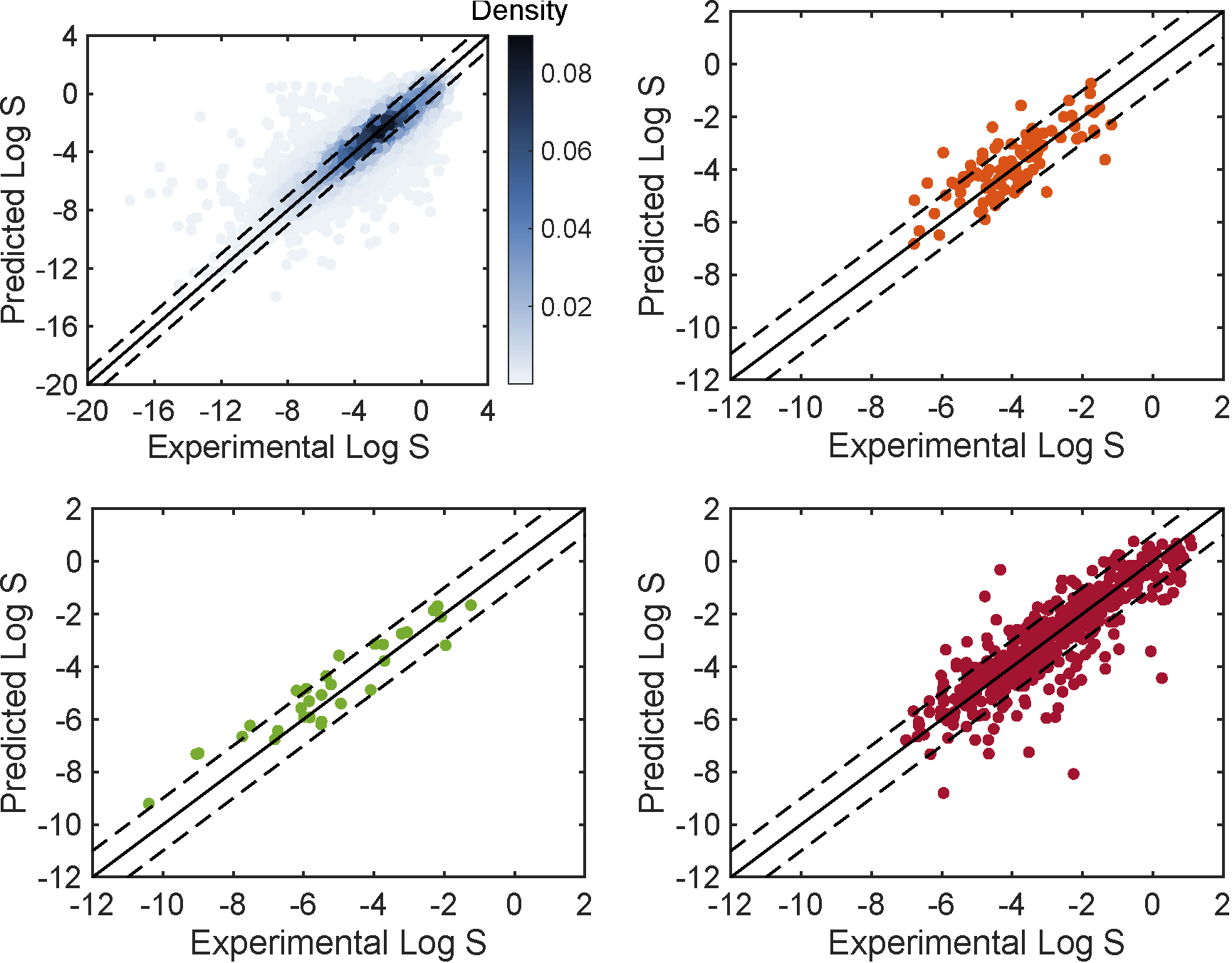


**Figure S11. Correlation plots for GNN.**


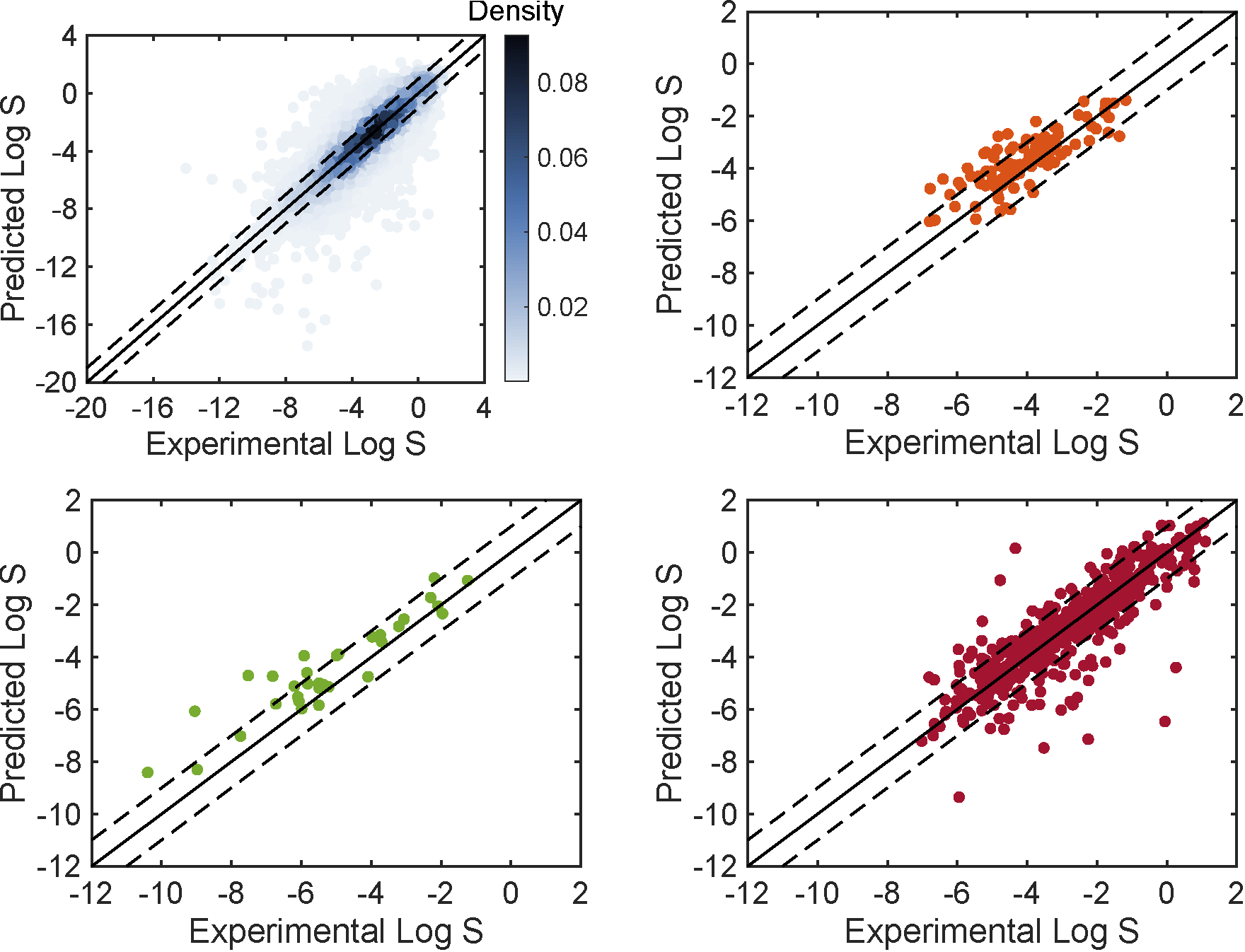


**Figure S12. Correlation plots for Transformer-CNN.**

# Supplementary Note 7: Correlation between predictions by different models and the SD among the Stacking models’ predictions


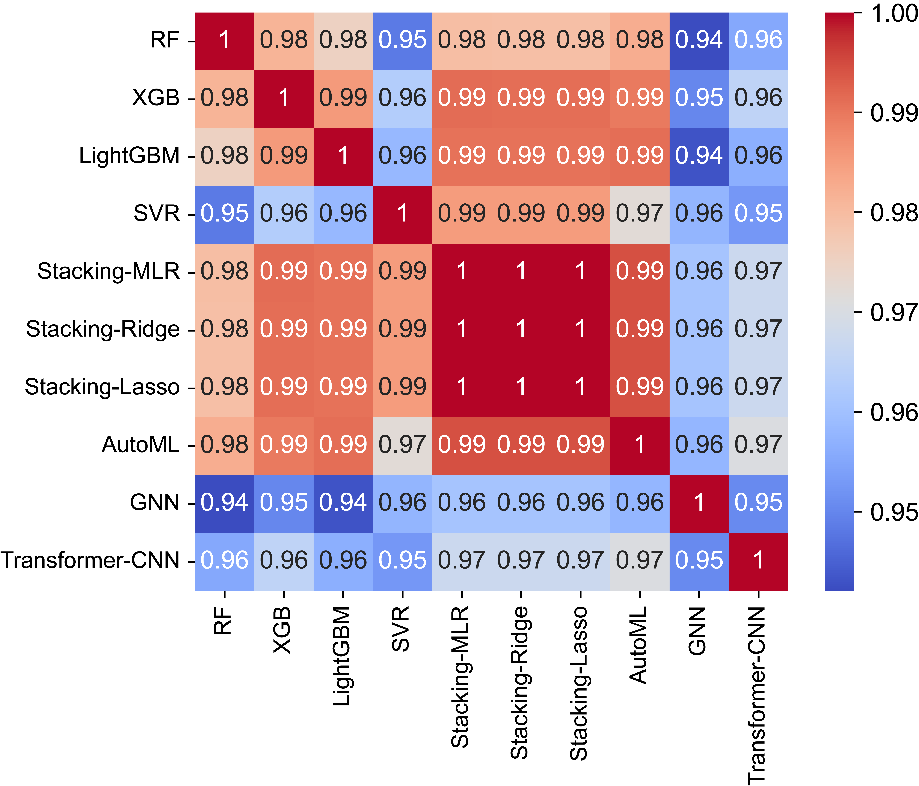


**Figure S13. Correlation between predictions by different models.**


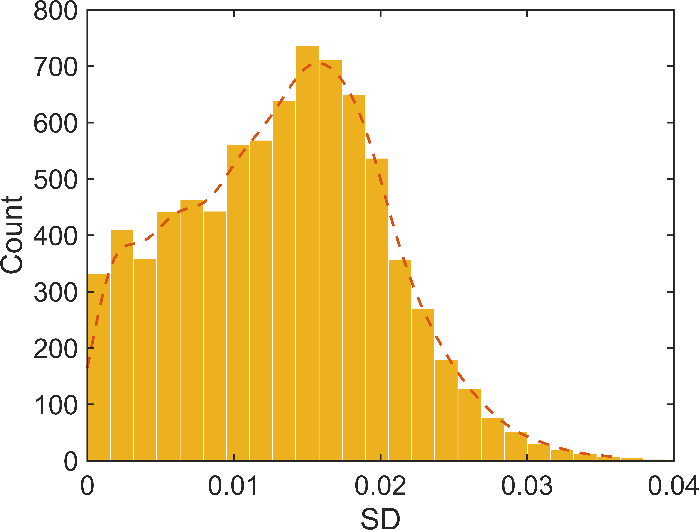


**Figure S14. SD among the Stacking models’ predictions.**

# Supplementary Note 8: Dotted SHAP figures

**
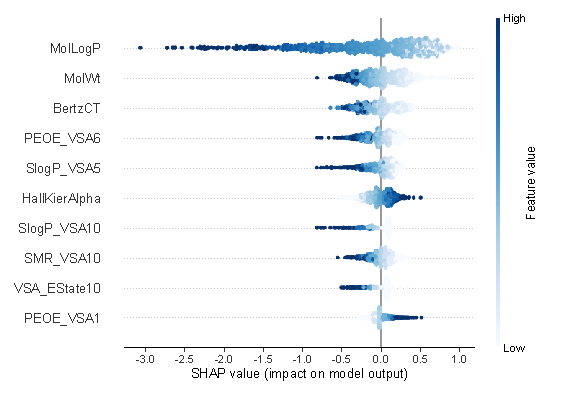
**

**Figure S15. Dotted beeswarm SHAP plot.**

**
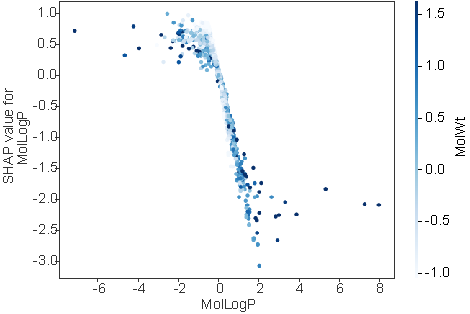
**

**Figure S16. Scatter plot of the relationship between SHAP values and MolLogP.**

# Supplementary Note 9: Hyperparameter optimization for classification tasks

A grid search method is utilized with 5-fold cross-validation on the CASR-2 dataset to determine the optimal hyperparameters for all algorithms. Stacking models are constructed using base learners with their optimized hyperparameters. For stacking models, the hyperparameters of the meta-learners are also specified. The optimal hyperparameters are summarized below:

**Table S9. Optimized hyperparameters in classification.**

| **Model** | **Hyperparameter** | **Value** |
| --- | --- | --- |
| RF | n_estimators | 400 |
|  | max_depth | 45 |
| XGB | n_estimators | 300 |
|  | max_depth | 13 |
|  | learning_rate | 0.08 |
| LightGBM | n_estimators | 500 |
|  | max_depth | 5 |
|  | learning_rate | 0.1 |
|  | num_leaves | 30 |
| SVR | kernel | ‘rbf’ |
|  | C | 1 |
|  | gamma | 0.07 |
| Stacking-LR | fit_intercept | False |
|  | C | 0.3 |

# Supplementary Note 10: Detailed classification results

**Table S10. Classification on internal set by 5-fold cross-validation^a), b)^.**

| **Method** | **Accuracy** | **Precision** | **Recall** | **F1** | **AUC** | **AP** | **Classification score** |
| --- | --- | --- | --- | --- | --- | --- | --- |
| RF | 0.651 | 0.538 | 0.466 | 0.480 | 0.893 | 0.532 | 0 |
| XGB | 0.645 | 0.520 | **0.475** | **0.487** | 0.890 | 0.519 | 2 |
| Lighjtgbm | 0.640 | 0.514 | 0.469 | 0.482 | 0.884 | 0.502 | 0 |
| SVC | 0.617 | 0.509 | 0.415 | 0.419 | 0.875 | 0.464 | 0 |
| Stacking-LR | **0.654** | **0.573** | 0.448 | 0.450 | **0.897** | **0.533** | **4** |
| AutoML | / | / | / | / | / | / | / |

^a)^Total results for 5-fold cross-validation; ^b)^Best value for each metric is in **bold**.

**Table S11. Classification performance on external sets^a), b)^.**

| **Database** | **Method** | **Metrics** | | | | | | |
| --- | --- | --- | --- | --- | --- | --- | --- | --- |
|  |  | **Accuracy** | **Precision** | **Recall** | **F1** | **AUC** | **AP** | **Classification score** |
| **SC2-1** | RF | 0.726 | 0.569 | 0.719 | 0.618 | 0.889 | 0.718 | 0 |
|  | XGB | 0.700 | 0.554 | 0.707 | 0.605 | 0.884 | 0.635 | 0 |
|  | LightGBM | 0.720 | 0.574 | **0.736** | 0.627 | 0.890 | 0.722 | 1 |
|  | SVR | 0.720 | 0.439 | 0.472 | 0.452 | 0.876 | 0.520 | 0 |
|  | Stacking-LR | **0.728** | 0.575 | 0.723 | 0.624 | **0.896** | **0.763** | **3** |
|  | AutoML | 0.720 | **0.600** | 0.727 | **0.645** | 0.886 | 0.706 | 2 |
| **SC2-2** | RF | 0.888 | 0.781 | 0.832 | 0.800 | 0.941 | 0.786 | 0 |
|  | XGB | 0.906 | 0.812 | 0.897 | 0.847 | 0.947 | 0.813 | 0 |
|  | LightGBM | **0.938** | **0.854** | **0.907** | **0.876** | **0.960** | **0.845** | **6** |
|  | SVR | 0.812 | 0.417 | 0.480 | 0.434 | **0.960** | 0.791 | 1 |
|  | Stacking-LR | 0.875 | 0.772 | 0.828 | 0.791 | 0.956 | 0.790 | 0 |
|  | AutoML | 0.906 | 0.812 | 0.897 | 0.847 | 0.951 | 0.841 | 0 |
| **DrugBank** | RF | 0.650 | 0.608 | 0.539 | 0.550 | 0.894 | 0.585 | 0 |
|  | XGB | 0.654 | 0.614 | 0.548 | 0.560 | 0.888 | 0.583 | 0 |
|  | LightGBM | **0.664** | 0.619 | **0.570** | **0.578** | 0.893 | 0.580 | **3** |
|  | SVR | 0.566 | 0.493 | 0.388 | 0.375 | 0.870 | 0.476 | 0 |
|  | Stacking-LR | 0.646 | 0.636 | 0.519 | 0.534 | **0.901** | 0.589 | 1 |
|  | AutoML | 0.649 | **0.646** | 0.511 | 0.528 | 0.900 | **0.593** | 2 |
| **All** | RF | 0.675 | 0.611 | 0.584 | 0.574 | 0.896 | 0.617 | 0 |
|  | XGB | 0.675 | 0.615 | 0.592 | 0.582 | 0.890 | 0.603 | 0 |
|  | LightGBM | **0.687** | 0.624 | **0.615** | **0.602** | 0.896 | 0.617 | **3** |
|  | SVR | 0.604 | 0.480 | 0.406 | 0.391 | 0.876 | 0.500 | 0 |
|  | Stacking-LR | 0.671 | 0.633 | 0.568 | 0.562 | **0.903** | **0.628** | 2 |
|  | AutoML | 0.674 | **0.647** | 0.566 | 0.564 | 0.900 | 0.624 | 1 |

^a)^Total results of five repetitions; ^b)^Best value for each metric is in **bold**.


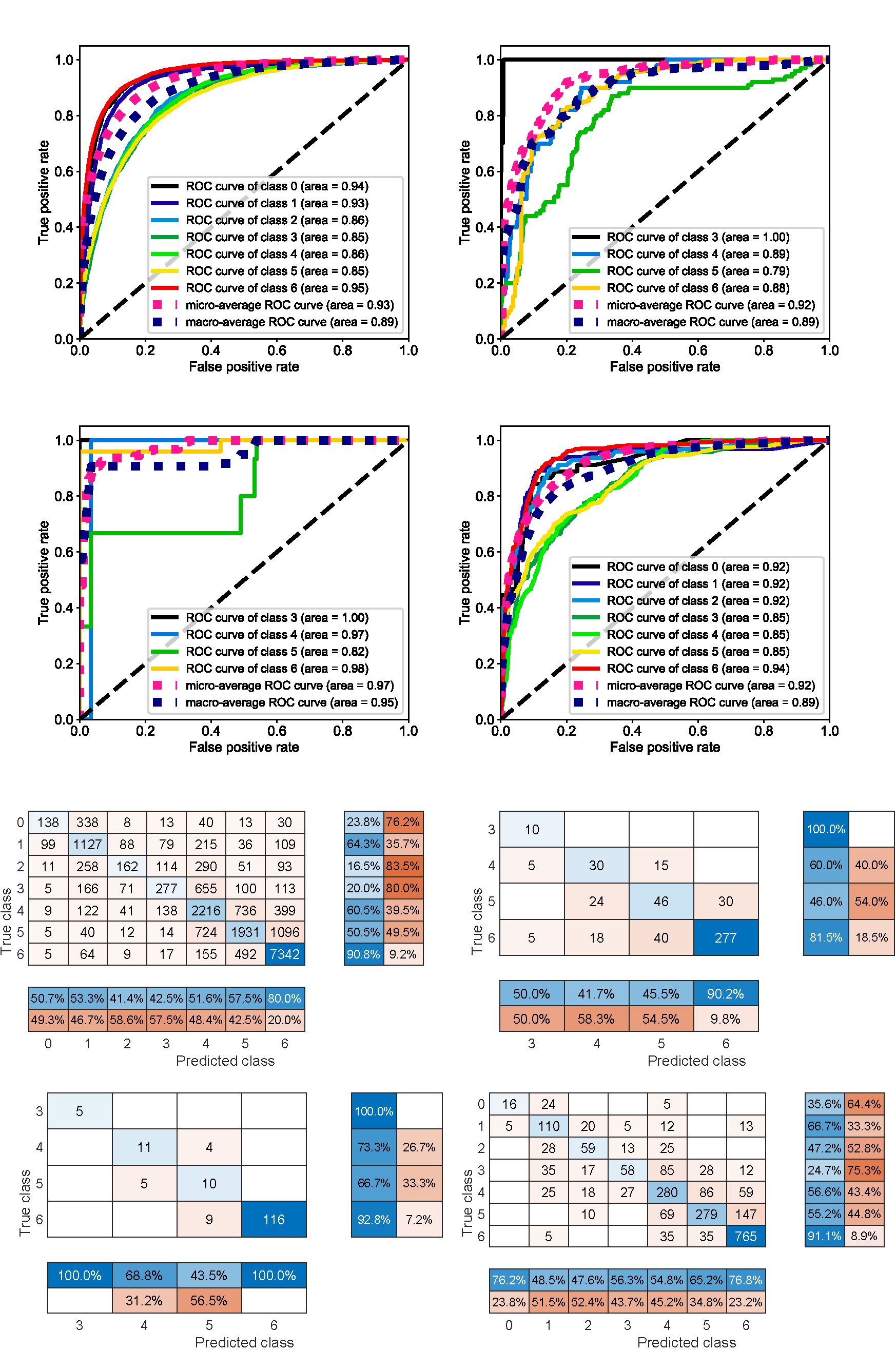


**Figure S17. ROC curves and CMs for RF.**


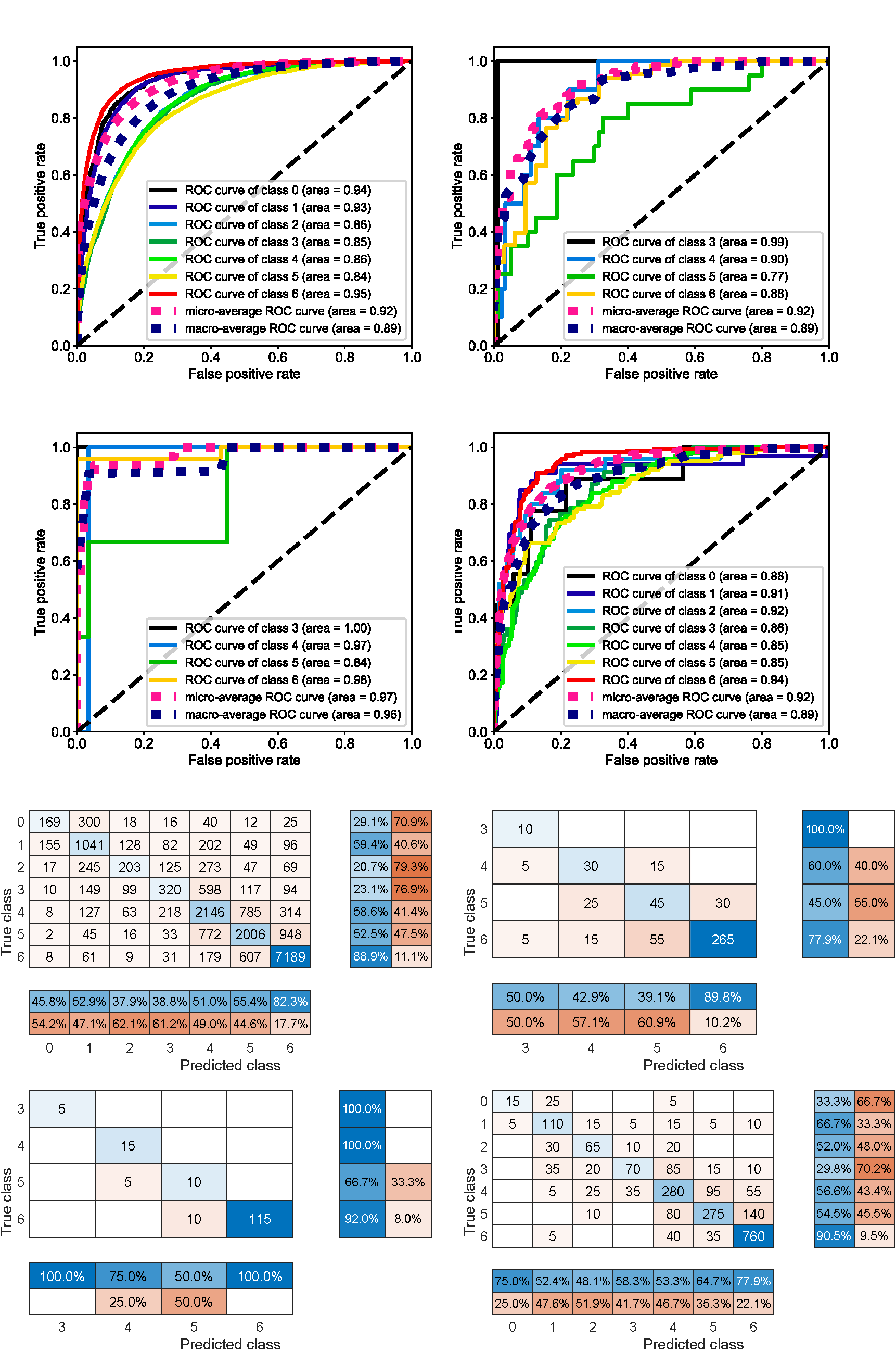


**Figure S18. ROC curves and CMs for XGB.**


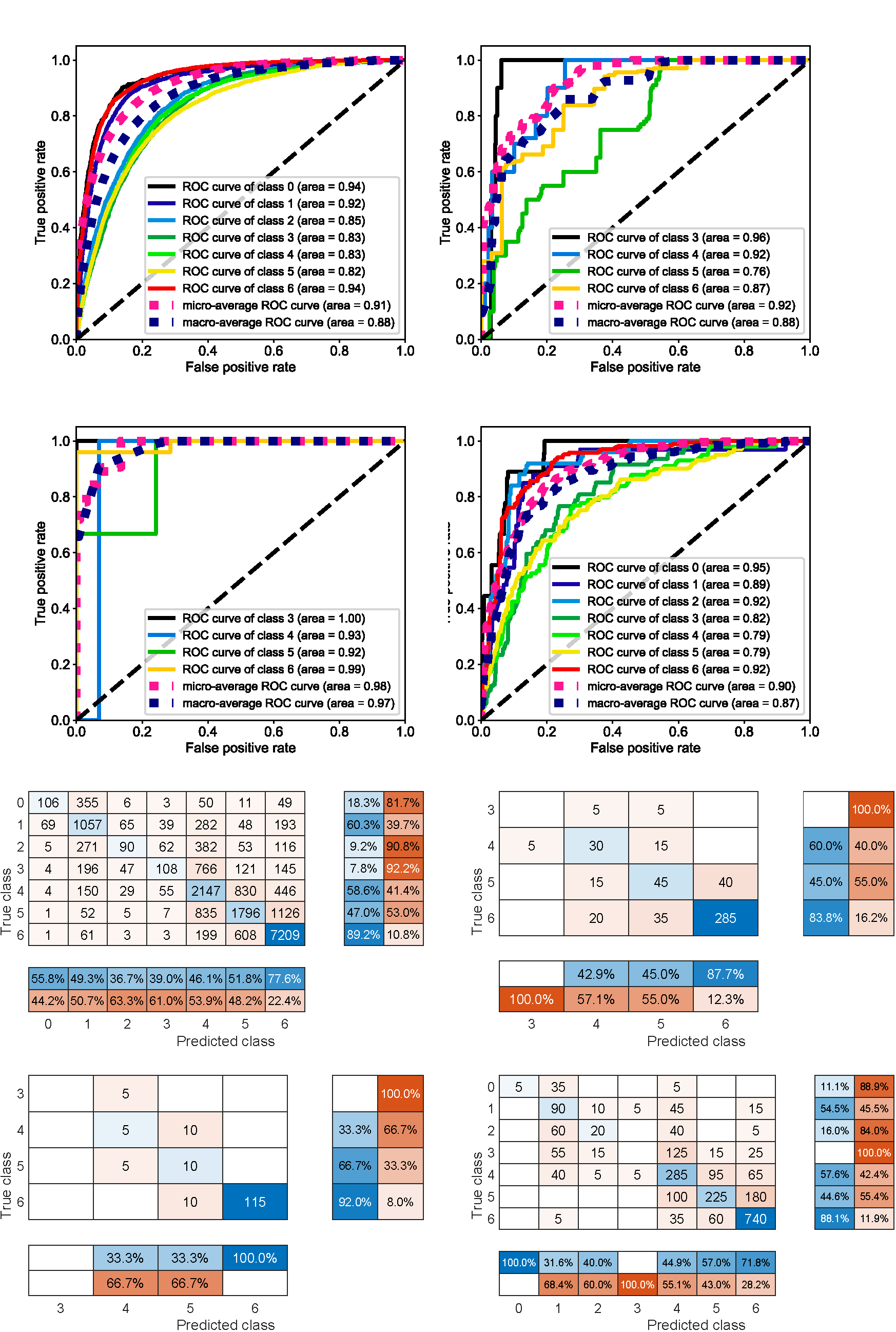


**Figure S19. ROC curves and CMs for SVC.**


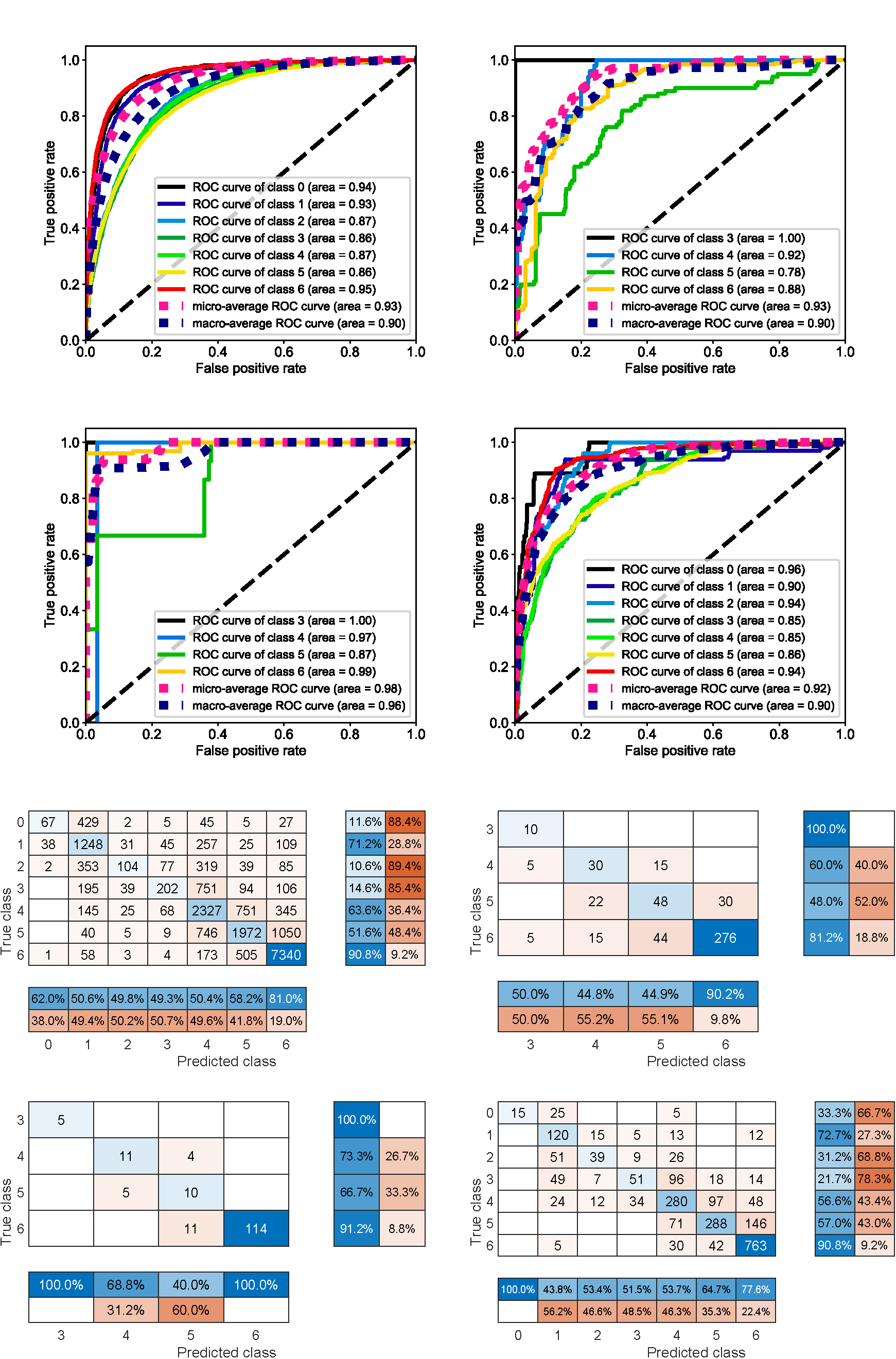


**Figure S20. ROC curves and CMs for Stacking-LR.**


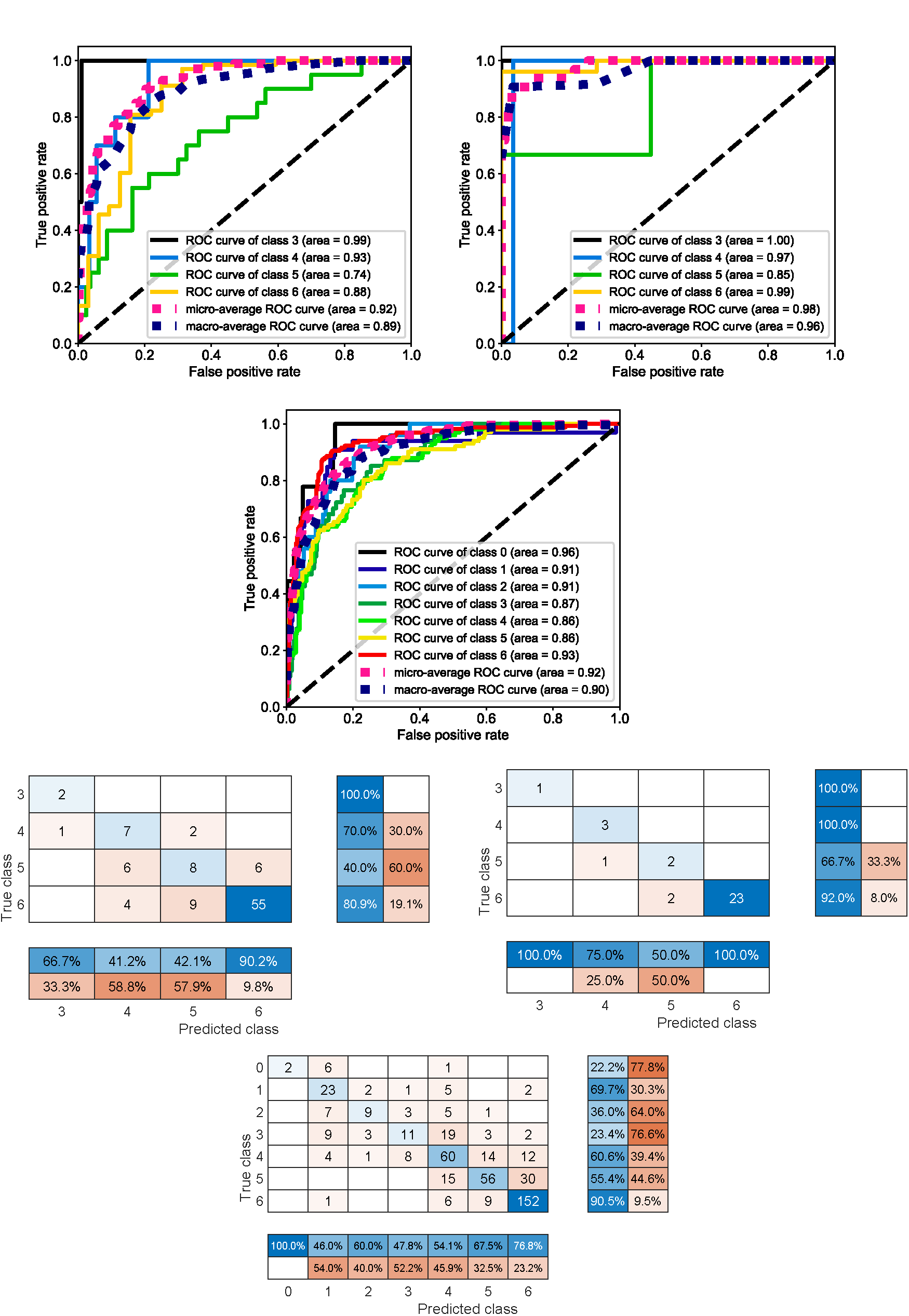


**Figure S21. ROC curves and CMs for AutoML.**

# Supplementary Note 11: Detailed results of applying data re-sampling techniques

In the main article, the results are presented as a spider plot to enhance the clarity of the figure. However, only the accuracy metric is displayed, while the other five metrics are omitted for simplicity. Detailed information, including all metrics, is provided below. It is important to note that the test set for each fold remains unaffected by data re-sampling.

**Table S12. Effect of data re-sampling, metrics are total results of cross-validations.**

| **Model** | **Technique** | **Metrics** | | | | | |
| --- | --- | --- | --- | --- | --- | --- | --- |
|  |  | **Accuracy** | **Precision** | **Recall** | **F1** | **AUC** | **AP** |
| RF | SMOTE | 0.6412 | 0.5027 | 0.4971 | 0.4991 | 0.8857 | 0.5232 |
|  | RandomOverSampler | 0.6492 | 0.5222 | 0.4849 | 0.4955 | 0.8872 | 0.5272 |
|  | SMOTEENN | 0.5817 | 0.4623 | 0.4865 | 0.4692 | 0.8650 | 0.4832 |
|  | BorderlineSMOTE-borderline-1 | 0.6414 | 0.5088 | 0.4958 | 0.5002 | 0.8868 | 0.5237 |
|  | BorderlineSMOTE-borderline-2 | 0.6419 | 0.5100 | 0.4859 | 0.4929 | 0.8852 | 0.5160 |
|  | RandomUnderSampler | 0.5510 | 0.4315 | 0.4702 | 0.4369 | 0.8504 | 0.4525 |
| XGB | SMOTE | 0.6425 | 0.5036 | 0.4889 | 0.4941 | 0.8841 | 0.5132 |
|  | RandomOverSampler | 0.6443 | 0.5100 | 0.4844 | 0.4929 | 0.8851 | 0.5156 |
|  | SMOTEENN | 0.5803 | 0.4562 | 0.4777 | 0.4626 | 0.8588 | 0.4698 |
|  | BorderlineSMOTE-borderline-1 | 0.6461 | 0.5126 | 0.4954 | 0.5016 | 0.8852 | 0.5168 |
|  | BorderlineSMOTE-borderline-2 | 0.6436 | 0.5052 | 0.4876 | 0.4938 | 0.8846 | 0.5103 |
|  | RandomUnderSampler | 0.5274 | 0.4060 | 0.4466 | 0.4131 | 0.8334 | 0.4164 |
| LightGBM | SMOTE | 0.6344 | 0.4959 | 0.4812 | 0.4860 | 0.8781 | 0.4944 |
|  | RandomOverSampler | 0.6301 | 0.4922 | 0.4754 | 0.4814 | 0.8795 | 0.4943 |
|  | SMOTEENN | 0.5777 | 0.4520 | 0.4734 | 0.4592 | 0.8596 | 0.4536 |
|  | BorderlineSMOTE-borderline-1 | 0.6350 | 0.4999 | 0.4830 | 0.4887 | 0.8781 | 0.4909 |
|  | BorderlineSMOTE-borderline-2 | 0.6321 | 0.4957 | 0.4805 | 0.4856 | 0.8783 | 0.4908 |
|  | RandomUnderSampler | 0.5097 | 0.3968 | 0.4362 | 0.4032 | 0.8278 | 0.4033 |
| SVC | SMOTE | 0.5939 | 0.4594 | 0.4728 | 0.4632 | 0.8657 | 0.4589 |
|  | RandomOverSampler | 0.5954 | 0.4627 | 0.4742 | 0.4659 | 0.8686 | 0.4604 |
|  | SMOTEENN | 0.5485 | 0.4301 | 0.4590 | 0.4352 | 0.8461 | 0.4246 |
|  | BorderlineSMOTE-borderline-1 | 0.5978 | 0.4631 | 0.4670 | 0.4639 | 0.8669 | 0.4572 |
|  | BorderlineSMOTE-borderline-2 | 0.5916 | 0.4607 | 0.4685 | 0.4629 | 0.8669 | 0.4540 |
|  | RandomUnderSampler | 0.5134 | 0.3867 | 0.4262 | 0.3940 | 0.8372 | 0.4049 |
| Stacking-LR | SMOTE | 0.6422 | 0.5109 | 0.4769 | 0.4879 | 0.8509 | 0.5034 |
|  | RandomOverSampler | 0.6318 | 0.5473 | 0.4450 | 0.4659 | 0.8774 | 0.5137 |
|  | SMOTEENN | 0.5970 | 0.4662 | 0.4771 | 0.4698 | 0.8228 | 0.4691 |
|  | BorderlineSMOTE-borderline-1 | 0.6502 | 0.5249 | 0.4860 | 0.4977 | 0.8617 | 0.5070 |
|  | BorderlineSMOTE-borderline-2 | 0.6435 | 0.5185 | 0.4712 | 0.4841 | 0.8814 | 0.5164 |
|  | RandomUnderSampler | 0.5386 | 0.4139 | 0.4616 | 0.4106 | 0.8582 | 0.4380 |

# Supplementary Note 12: Detailed results of applying data re-sampling technique

**Table S13. Performance comparison of the Stacking-Lasso model and other reference models for solubility prediction on 10 experimentally validated potential drugs..**

| **Method** | **RMSE** | **R^2^** | **%Log S ± 0.7** | **%Log S ± 1.0** | **MAPD** |
| --- | --- | --- | --- | --- | --- |
| **This work** | **0.436** | **0.917** | **90.0** | **90.0** | **29.2** |
| ALOGPS | 0.633 | 0.694 | 80.0 | 90.0 | 67.3 |
| GSE | 1.056 | 0.237 | 70.0 | 70.0 | 93.1 |
| ASE | 3.300 | -3.942 | 30.0 | 40.0 | 184.0 |
| Modified ASE | 0.935 | 0.603 | 60.0 | 70.0 | 106.6 |

# Supplementary Note 13: Examples of discarded ambiguous entries of DrugBank database

**Table S14. Examples of discarded ambiguous entries of DrugBank database.**

| **CAS-RN** | **SMILES** | **Experimental solubility** |
| --- | --- | --- |
| 54-47-7 | CC1=NC=C(COP(O)(O)=O)C(C=O)=C1O | Appreciable |
| 79490-84-9 | CCC(NC(C)C)C(O)C1=CC(O)=C(O)C=C1 | Appreciable |
| 1695-77-8 | [H][C@@]12O[C@H](C)CC(=O)[C@]1(O)O[C@]1([H])[C@@H](NC)[C@@H](O)[C@@H](NC)[C@H](O)[C@@]1([H])O2 | Easily soluble in cold water |
| 62013-04-1 | CC[C@H]1OC(=O)[C@H](C)[C@@H](O[C@H]2C[C@@](C)(OC)[C@@H](O)[C@H](C)O2)[C@H](C)[C@@H](O[C@@H]2O[C@H](C)C[C@@H]([C@H]2O)N(C)C)[C@](C)(O)C[C@@H](C)[C@@H]2N[C@@H](COCCOC)O[C@H]([C@H]2C)[C@]1(C)O | Poor |
| 137234-62-9 | C[C@@H](C1=NC=NC=C1F)[C@](O)(CN1C=NC=N1)C1=C(F)C=C(F)C=C1 | Low |
| 42408-82-2 | [H][C@@]12CC3=C(C=C(O)C=C3)[C@]3(CCCC[C@@]13O)CCN2CC1CCC1 | Moderate |
| 864750-70-9 | CN(CCN1CCC(CC1)OC(=O)NC1=CC=CC=C1C1=CC=CC=C1)C(=O)C1=CC=C(CN2CCC(CC2)C(N)=O)C=C1 | < 1 mg/ml |
| 71-44-3 | NCCCNCCCCNCCCN | > 100 mg/mL |
| 1477-40-3 | CC[C@H](OC(C)=O)C(C[C@H](C)N(C)C)(C1=CC=CC=C1)C1=CC=CC=C1 | >15 mg/mL |
| 594839-88-0 | OC(=O)C1=CC=C2N=C(OC2=C1)C1=CC(Cl)=CC(Cl)=C1 | >50mg/mL |

# Supplementary Note 14: Conversion of solubility values and the corresponding classes

**Table S15. Conversion of solubility values and the corresponding classes.**^[12]^

| **S (g/L) range** | **Class** | **Amount of water (mL) required to dissolve 1 g of drug** |
| --- | --- | --- |
| > 1000 | Very soluble (0) | < 1 |
| 100-1000 | Freely soluble (1) | 1-10 |
| 33-100 | Soluble (2) | 10-30 |
| 10-33 | Sparingly soluble (3) | 30-100 |
| 1-10 | Slightly soluble (4) | 100-1000 |
| 0.1-1 | Very slightly soluble (5) | 1000-10000 |
| < 0.1 | Practically insoluble or insoluble (6) | > 10000 |

# Supplementary Note 15: Dimension reduction

The t-SNE technique is applied to reduce the dimensionality of the descriptor space to two dimensions. Unlike PCA, t-SNE is a nonlinear method capable of effectively capturing similarities among data points. A perplexity value of 50 is used in this process, as a relatively large perplexity is required to handle a substantial dataset and to group the data into meaningful clusters. This value falls within the recommended range of 5 to 50 for perplexity.

# Supplementary Note 16: Applicability domain analysis

**Euclidean distance method**

The Euclidean distance between a molecule 𝑖 and a molecule 𝑗 in the training set is calculated as:

$$\begin{aligned} d_{ij}= \sqrt{{(D_{i1}- D_{j1})}^{2}+\ldots+ {(D_{iK}- D_{jK})}^{2}}\#\left( 8 \right) \end{aligned}$$

where D represents the standardized features, and K is the number of features.

The average distance of molecule 𝑖 to the molecules in the training set is given by:

$$\begin{aligned} d_{i}= \frac{1}{T} \sum_{j=1}^{T} d_{ij}\#\left( 9 \right) \end{aligned}$$

where T is the total number of molecules in the training set.

**Probability density method**

The probability density of each point in the training set and within the entire mesh grid is estimated using a normal kernel function. The 95% highest density region (HDR) is determined by selecting the 95% of points with the highest density values from the training set. This approach avoids the computational complexity of general Monte Carlo integration methods.

# Supplementary Note 17: General solubility equation and Abraham solubility equation

In 1980 Yalkowsky et al.^[13]^ combined the previous findings to develop the GSE and estimate the aqueous solubility of non-electrolytes using the melting point (MP) and Log P:

$$\begin{aligned} Log S=0.5-0.01 \times\left( MP-25 \right)-Log P\#\left( 10 \right) \end{aligned}$$

The original ASE was proposed by Prof. Michael Abraham and his coworker^[14]^:

$$\begin{aligned} LogS=0.518-1.004E+0.771S+2.168A+4.238B-3.362A\times B-3.987V\# (11) \end{aligned}$$

where A is the sum of H-bond acidity, B is the sum of H-bond basicity, S is the dipolarity/polarizability, E is an excess molar refraction, and V is the McGowan characteristic volume. To fully explore the potential of ASE model, we refer to the work of Prof. Avdeef^[5]^ and recalibrate the coefficients of the ASE using our training set of about 20k data. The R^2^ is substantially increased from -3.702 to 0.421, giving the modified version of ASE as:

$$\begin{aligned} Log S=-1.545-1.031E-0.085S+1.119A+1.870B-0.166A\times B-1.170V\#\left( 12 \right) \end{aligned}$$

# Supplementary Note 18: COSMO-RS calculations

**Table S16. Molecules with calculation failure in structure optimization.**

| Name | SMILES |
| --- | --- |
| Altretamine | CN(C)C1=NC(=NC(=N1)N(C)C)N(C)C |
| Aminophylline | CN1C2=C(C(=O)N(C1=O)C)NC=N2.CN1C2=C(C(=O)N(C1=O)C)NC=N2.C(CN)N |
| Anisotropine methylbromide | CCCC(CCC)C(=O)OC1C[C@H]2CC[C@@H](C1)[N+]2(C)C.[Br-] |
| Brilliant Blue G | CCN(CC1=CC(=CC=C1)S(=O)(=O)[O-])C2=CC(=C(C=C2)/C(=C/3\C=CC(=[N+](CC)CC4=CC(=CC=C4)S(=O)(=O)[O-])C=C3C)/C5=CC=C(C=C5)NC6=CC=C(C=C6)OCC)C.[Na+] |
| Calcium lactate | CC(C(=O)[O-])O.CC(C(=O)[O-])O.[Ca+2] |
| Carbenicillin | CC1([C@@H](N2[C@H](S1)[C@@H](C2=O)NC(=O)C(C3=CC=CC=C3)C(=O)O)C(=O)O)C |
| Cefepime | C[N+]1(CCCC1)CC2=C(N3[C@@H]([C@@H](C3=O)NC(=O)/C(=N\OC)/C4=CSC(=N4)N)SC2)C(=O)[O-] |
| Cefprozil | C/C=C/C1=C(N2[C@@H]([C@@H](C2=O)NC(=O)[C@@H](C3=CC=C(C=C3)O)N)SC1)C(=O)O |
| Chlormerodrin | COC(CNC(=O)N)C[Hg]Cl |
| Clarithromycin | CC[C@@H]1[C@@]([C@@H]([C@H](C(=O)[C@@H](C[C@@]([C@@H]([C@H]([C@@H]([C@H](C(=O)O1)C)O[C@H]2C[C@@]([C@H]([C@@H](O2)C)O)(C)OC)C)O[C@H]3[C@@H]([C@H](C[C@H](O3)C)N(C)C)O)(C)OC)C)C)O)(C)O |
| Cortisone Acetate | CC(=O)OCC(=O)[C@]1(CC[C@@H]2[C@@]1(CC(=O)[C@H]3[C@H]2CCC4=CC(=O)CC[C@]34C)C)O |
| Digitoxin | C[C@@H]1[C@H]([C@H](C[C@@H](O1)O[C@@H]2[C@H](O[C@H](C[C@@H]2O)O[C@@H]3[C@H](O[C@H](C[C@@H]3O)O[C@H]4CC[C@]5([C@@H](C4)CC[C@@H]6[C@@H]5CC[C@]7([C@@]6(CC[C@@H]7C8=CC(=O)OC8)O)C)C)C)C)O)O |
| Diiodotyrosine | C1=C(C=C(C(=C1I)O)I)C[C@@H](C(=O)O)N |
| Dimenhydrinate | CN1C2=C(C(=O)N(C1=O)C)NC(=N2)Cl.CN(C)CCOC(C1=CC=CC=C1)C2=CC=CC=C2 |
| Erythromycin | CC[C@@H]1[C@@]([C@@H]([C@H](C(=O)[C@@H](C[C@@]([C@@H]([C@H]([C@@H]([C@H](C(=O)O1)C)O[C@H]2C[C@@]([C@H]([C@@H](O2)C)O)(C)OC)C)O[C@H]3[C@@H]([C@H](C[C@H](O3)C)N(C)C)O)(C)O)C)C)O)(C)O |
| Ethotoin | CCN1C(=O)C(NC1=O)C2=CC=CC=C2 |
| Idoxuridine | C1[C@@H]([C@H](O[C@H]1N2C=C(C(=O)NC2=O)I)CO)O |
| Levothyroxine | C1=C(C=C(C(=C1I)OC2=CC(=C(C(=C2)I)O)I)I)C[C@@H](C(=O)O)N |
| Meprobamate | CCCC(C)(COC(=O)N)COC(=O)N |
| Mycostatin | C[C@H]1/C=C/C=C/CC/C=C/C=C/C=C/C=C/C(CC2C(C(C[C@](O2)(CC(C(CCC(CC(CC(CC(=O)O[C@H]([C@@H]([C@@H]1O)C)C)O)O)O)O)O)O)O)C(=O)O)O[C@@H]3[C@H]([C@H]([C@@H]([C@H](O3)C)O)N)O |
| Oritavancin | C[C@H]1[C@@H]([C@@](C[C@@H](O1)O[C@H]2[C@H]3C(=O)N[C@@H](C4=C(C(=CC(=C4)O)O)C5=C(C=CC(=C5)[C@H](C(=O)N3)NC(=O)[C@H]6C7=CC(=C(C(=C7)OC8=C(C=C2C=C8)Cl)O[C@H]9[C@@H]([C@H]([C@@H]([C@H](O9)CO)O)O)O[C@H]1C[C@]([C@H]([C@@H](O1)C)O)(C)NCC1=CC=C(C=C1)C1=CC=C(C=C1)Cl)OC1=C(C=C(C=C1)[C@H]([C@H](C(=O)N[C@H](C(=O)N6)CC(=O)N)NC(=O)[C@@H](CC(C)C)NC)O)Cl)O)C(=O)O)(C)N)O |
| Pancuronium | CC(=O)O[C@H]1C[C@@H]2CC[C@@H]3[C@@H]([C@]2(C[C@@H]1N4CCCCC4)C)CC[C@]5([C@H]3C[C@@H]([C@@H]5OC(=O)C)[N+]6(CCCCC6)C)C.[Br-] |
| Picrotoxin | CC(=C)[C@@H]1C2[C@@H]3[C@@]4([C@](C1C(=O)O2)(CC5[C@]4(O5)C(=O)O3)O)C.C[C@@]12[C@H]3C4[C@H](C([C@@]1(C[C@@H]5[C@]2(O5)C(=O)O3)O)C(=O)O4)C(C)(C)O |
| Rifabutin | C[C@H]1/C=C/C=C(\C(=O)N=C2C(=C3C(=C4C2=NC5(N4)CCN(CC5)CC(C)C)C6=C(C(=C3O)C)O[C@@](C6=O)(O/C=C/[C@@H]([C@H]([C@H]([C@@H]([C@@H]([C@@H]([C@H]1O)C)O)C)OC(=O)C)C)OC)C)O)/C |
| Rifampin | C[C@H]1/C=C/C=C(\C(=O)NC2=C(C(=C3C(=C2O)C(=C(C4=C3C(=O)[C@](O4)(O/C=C/[C@@H]([C@H]([C@H]([C@@H]([C@@H]([C@@H]([C@H]1O)C)O)C)OC(=O)C)C)OC)C)C)O)O)/C=N/N5CCN(CC5)C)/C |
| Sodium Citrate | C(C(=O)[O-])C(CC(=O)[O-])(C(=O)[O-])O.[Na+].[Na+].[Na+] |
| Sodium Hyaluronate | CC(=O)N[C@@H]1[C@H]([C@@H]([C@H](O[C@H]1O)CO)O)O[C@H]2[C@@H]([C@H]([C@@H]([C@H](O2)C(=O)O)O[C@H]3[C@@H]([C@H]([C@@H]([C@H](O3)CO)O)O[C@H]4[C@@H]([C@H]([C@@H]([C@H](O4)C(=O)O)O)O)O)NC(=O)C)O)O.[Na+] |
| Telaprevir | CCC[C@@H](C(=O)C(=O)NC1CC1)NC(=O)[C@@H]2[C@H]3CCC[C@H]3CN2C(=O)[C@H](C(C)(C)C)NC(=O)[C@H](C4CCCCC4)NC(=O)C5=NC=CN=C5 |
| Thiocolchicoside | CC(=O)N[C@H]1CCC2=CC(=C(C(=C2C3=CC=C(C(=O)C=C13)SC)OC)OC)O[C@H]4[C@@H]([C@H]([C@@H]([C@H](O4)CO)O)O)O |
| Tpmpa | CP(=O)(C1=CCNCC1)O |
| beta-carotene | CC1=C(C(CCC1)(C)C)/C=C/C(=C/C=C/C(=C/C=C/C=C(/C=C/C=C(/C=C/C2=C(CCCC2(C)C)C)\C)\C)/C)/C |
| vitamin B12 | CC1=CC2=C(C=C1C)N(C=N2)[C@@H]3[C@@H]([C@@H]([C@H](O3)CO)OP(=O)([O-])O[C@H](C)CNC(=O)CC[C@@]4([C@H]([C@@H]5[C@]6([C@@]([C@@H](/C(=C(/C7=N/C(=C\C8=N/C(=C(\C4=N5)/C)/[C@H](C8(C)C)CCC(=O)N)/[C@H]([C@]7(C)CC(=O)N)CCC(=O)N)\C)/[N-]6)CCC(=O)N)(C)CC(=O)N)C)CC(=O)N)C)O.[C-]#N.[Co+3] |

**Code example for solubility calculation in COSMOthermX:**

Results of non-iterative method is obtained along with iterative method.

**SLE method**

CTD=BP_TZVP_22.ctd CDIR=" BIOVIA\COSMOtherm2022\COSMOthermX\..\COSMOtherm\CTDATA-FILES" LDIR="BIOVIA\COSMOtherm2022\COSMOthermX\..\licensefiles"

NDGF WCMN NOTEMPTY WTLN EHFILE

!! GENERATED BY COSMOTHERMX !!

F=Mirabegron.cosmo

F=h2o_c0.cosmo FDIR=" BIOVIA_2022.BIOVIA_2022.AM_COSMObase_2022\COSMObase2022\BP-TZVP-COSMO\"

TC=25.0 SOLUB=2 SOLVDENS=997 SLESOL FORCE_QSPR AMINO_CORR LSOLOUT_MOLL_LOG10

**Iterative method**

CTD=BP_TZVP_22.ctd CDIR=" BIOVIA\COSMOtherm2022\COSMOthermX\..\COSMOtherm\CTDATA-FILES" LDIR="BIOVIA\COSMOtherm2022\COSMOthermX\..\licensefiles"

NDGF WCMN NOTEMPTY WTLN EHFILE

!! GENERATED BY COSMOTHERMX !!

F=Chlorprothixene.cosmo

F=h2o_c0.cosmo FDIR=" BIOVIA_2022.BIOVIA_2022.AM_COSMObase_2022\COSMObase2022\BP-TZVP-COSMO\"

TC=25.0 SOLUB=2 SOLVDENS=997 ITERATIVE FORCE_QSPR AMINO_CORR LSOLOUT_MOLL_LOG10

# Supplementary Note 19: Molecules for experiments


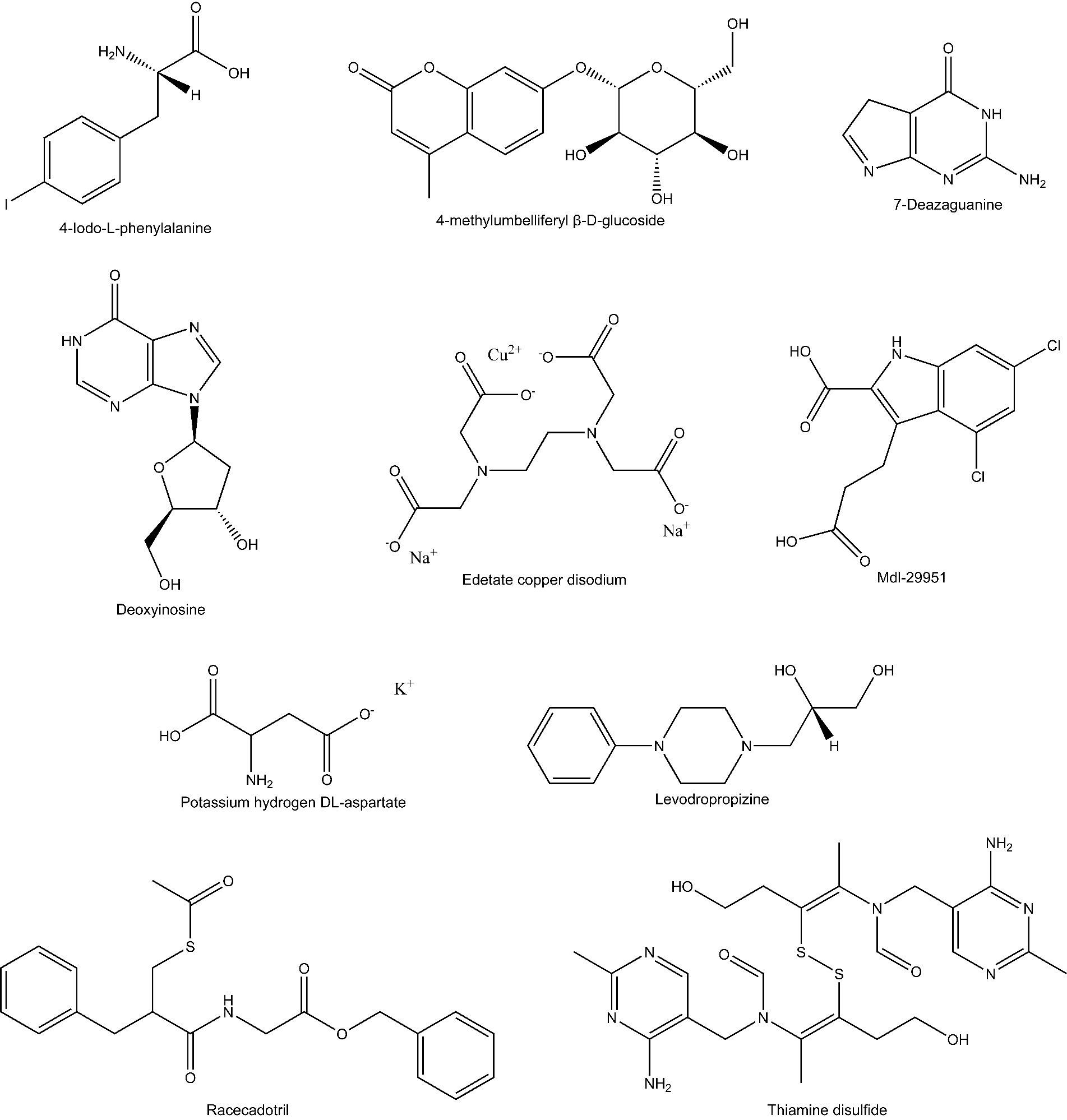


**Figure S22. Molecules for experiments.**

# Supplementary Note 20: An extremely insoluble (~ $\boldsymbol{1 \times}\boldsymbol{10}^{\boldsymbol{-6}} \mathbf{g/L}$) molecule in experiments

The predicted solubility of riboflavin tetrabutyrate is approximately $3.3 \times{10}^{-6} g/L$, which is challenging to measure using gravimetric methods or HPLC. To verify this prediction, ^1^H Nuclear Magnetic Resonance Spectra (^1^H NMR) spectroscopy was performed on a saturated aqueous solution of riboflavin tetrabutyrate using a Bruker Advance III instrument (Bruker, Switzerland). The spectrum showed only the peaks corresponding to water and deuterated water ($\delta=4.8 \mathrm{ppm}$), with no detectable solute signals. This observation confirms that the solute concentration is extremely low, supporting the accuracy of the model's prediction.


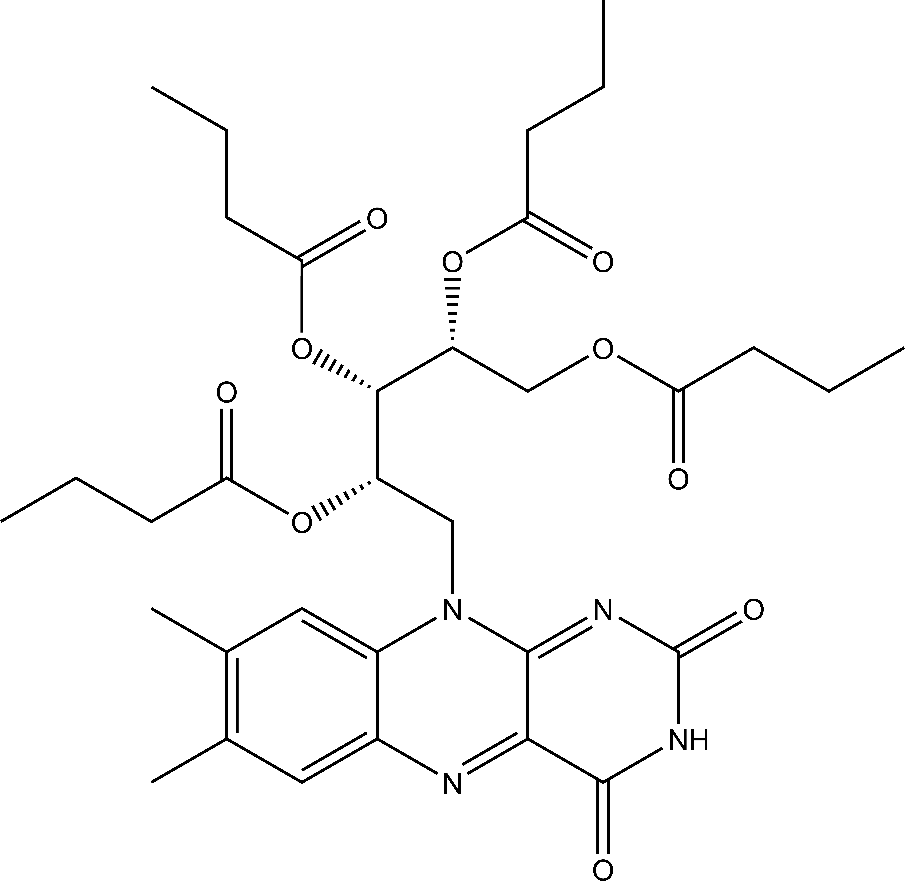


**Figure S23. Structure of Riboflavin tetrabutyrate.**


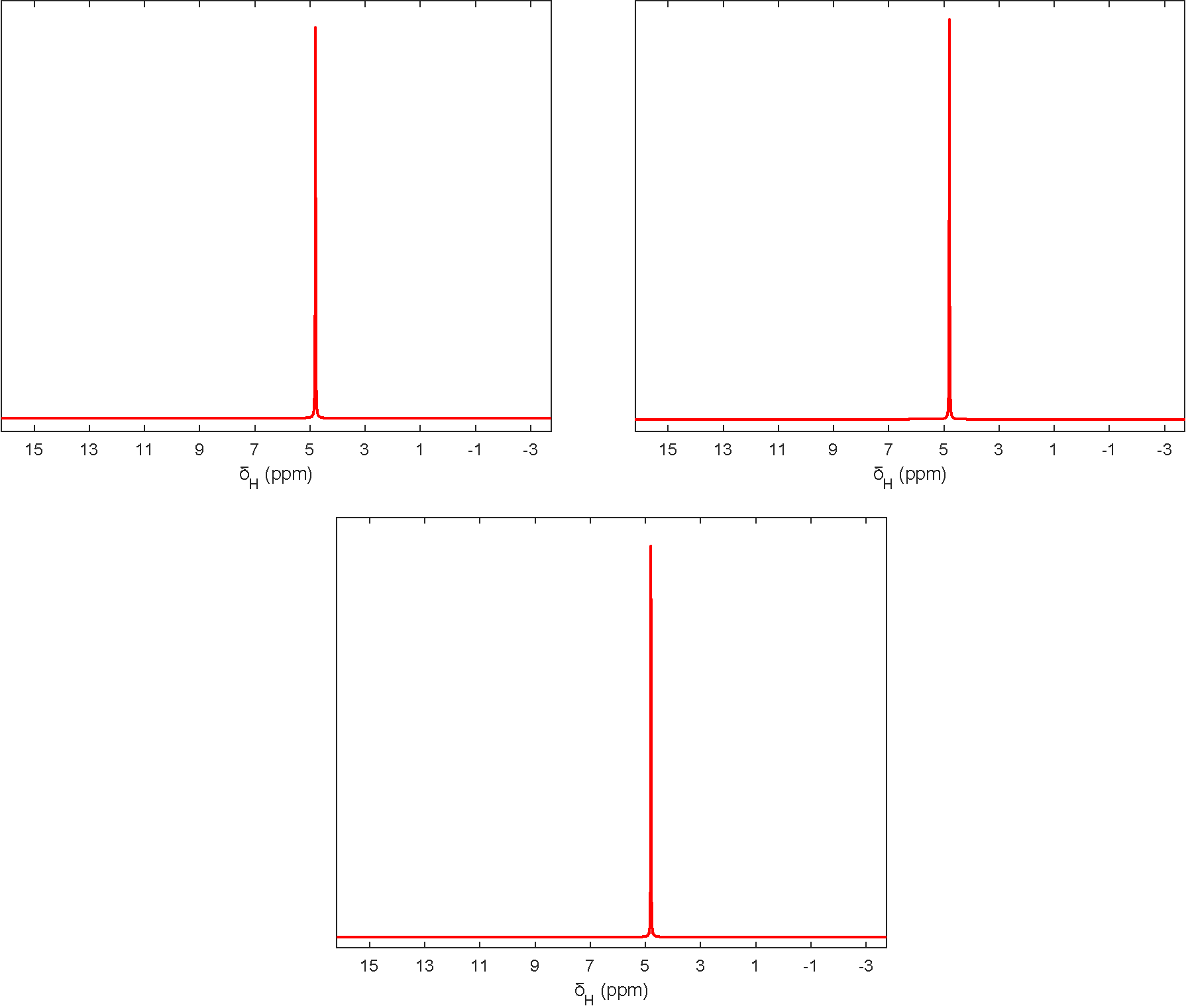


**Figure S24. HNMR results.**

# Supplementary Note 21: Principles for the selection of experimentally tested compounds

The molecules for experimental validation were chosen according to five guidelines: (1) Absence of experimentally solubility data. To ensure the rigorous evaluation of our model, the compounds will be excluded if solubility records have already existed in public databases; (2) Structural and solubility diversity. To comprehensively evaluate the generalization ability of our model, drugs with all solubility classes and different molecular types (e.g. salt, non-salt, and metal-containing compounds) are retained for further experiments; (3) Commercial availability. All drugs should be purchased from established commercial suppliers to ensure consistent quality; (4) Ambient-temperature stability. Compounds unstable at ambient conditions and requiring cryogenic storage are excluded to prevent decomposition of drugs that could lead to misleading results. (5) Group restriction. Only group A and B of the predicted reliability tiers by our model.

# References:

1. M. A. Ghanavati, S. Ahmadi, S. Rohani, A machine learning approach for the prediction of aqueous solubility of pharmaceuticals: A comparative model and dataset analysis. *Digital Discovery* 3, 2085-2104 (2024).

2. A. Tayyebi, A. S. Alshami, Z. Rabiei et al., Prediction of organic compound aqueous solubility using machine learning: a comparison study of descriptor-based and fingerprints-based models. *Journal of Cheminformatics* 15, 99 (2023).

3. T. Zhu, Y. Chen, C. Tao, Multiple machine learning algorithms assisted QSPR models for aqueous solubility: Comprehensive assessment with CRITIC-TOPSIS. *Science of the Total Environment* 857, 159448 (2023).

4. C. N. Lowe, N. Charest, C. Ramsland et al., Transparency in modeling through careful application of OECD’s QSAR/QSPR principles via a curated water solubility data set. *Chemical Research in Toxicology* 36, 465-478 (2023).

5. A. Avdeef, Prediction of aqueous intrinsic solubility of druglike molecules using Random Forest regression trained with Wiki-pS0 database. *ADMET and DMPK* 8, 29-77 (2020).

6. E. M. Tosca, R. Bartolucci, P. Magni, Application of artificial neural networks to predict the intrinsic solubility of drug-like molecules. *Pharmaceutics* 13, 1101 (2021).

7. M. C. Ramos, A. D. White, Predicting small molecules solubility on endpoint devices using deep ensemble neural networks. *Digital Discovery* 3, 786-795 (2024).

8. Q. Chen, Y. Zhang, P. Gao, J. Zhang, An interpretable graph representation learning model for accurate predictions of drugs aqueous solubility. *Artificial Intelligence Chemistry* 1, 100010 (2023).

9. G. Panapitiya, M. Girard, A. Hollas et al., Evaluation of deep learning architectures for aqueous solubility prediction. *ACS Omega* 7, 15695-15710 (2022).

10. Q. Cui, S. Lu, B. Ni et al., Improved prediction of aqueous solubility of novel compounds by going deeper with deep learning. *Frontiers in Oncology* 10, 121 (2020).

11. P. G. Francoeur, D. R. Koes, SolTranNet–A machine learning tool for fast aqueous solubility prediction. *Journal of Chemical Information and Modeling* 61, 2530-2536 (2021).

12. C. o. Europe, *European pharmacopoeia, 10th ed* (Council of Europe: Strasbourg, France, 2019), vol. 1.

13. S. H. Yalkowsky, S. C. Valvani, Solubility and partitioning I: solubility of nonelectrolytes in water. *Journal of Pharmaceutical Sciences* 69, 912-922 (1980).

14. M. H. Abraham, J. Le, The correlation and prediction of the solubility of compounds in water using an amended solvation energy relationship. *Journal of Pharmaceutical Sciences* 88, 868-880 (1999).
